# An LIR motif in the Rift Valley fever virus NSs protein is critical for the interaction with LC3 family members and inhibition of autophagy

**DOI:** 10.1371/journal.ppat.1012093

**Published:** 2024-03-21

**Authors:** Kaylee Petraccione, Mohamed G. H. Ali, Normand Cyr, Haytham M. Wahba, Timothy Stocker, Maryna Akhrymuk, Ivan Akhrymuk, Lauren Panny, Nicole Bracci, Raphaël Cafaro, Danuta Sastre, Andrew Silberfarb, Paul O’Maille, James Omichinski, Kylene Kehn-Hall

**Affiliations:** 1 Department of Biomedical Sciences and Pathobiology, Virginia-Maryland College of Veterinary Medicine, Virginia Polytechnic Institute and State University, Blacksburg, Virginia, United States of America; 2 Center for Emerging, Zoonotic, and Arthropod-borne Pathogens, Virginia Polytechnic Institute and State University, Blacksburg, Virginia, United States of America; 3 Department of Biochemistry and Molecular Medicine, Université de Montréal, Montréal, Quebec, Canada; 4 Department of Biochemistry, Faculty of Pharmacy, Beni-Suef University, Beni-Suef, Egypt; 5 Biosciences Division, SRI International, Menlo Park, California, United States of America; 6 Artificial Intelligence Center, SRI International, Menlo Park, California, United States of America; University of Pittsburgh, UNITED STATES

## Abstract

Rift Valley fever virus (RVFV) is a viral zoonosis that causes severe disease in ruminants and humans. The nonstructural small (NSs) protein is the primary virulence factor of RVFV that suppresses the host’s antiviral innate immune response. Bioinformatic analysis and AlphaFold structural modeling identified four putative LC3-interacting regions (LIR) motifs (NSs 1–4) in the RVFV NSs protein, which suggest that NSs interacts with the host LC3-family proteins. Using, isothermal titration calorimetry, X-ray crystallography, co-immunoprecipitation, and co-localization experiments, the C-terminal LIR motif (NSs4) was confirmed to interact with all six human LC3 proteins. Phenylalanine at position 261 (F261) within NSs4 was found to be critical for the interaction of NSs with LC3, retention of LC3 in the nucleus, as well as the inhibition of autophagy in RVFV infected cells. These results provide mechanistic insights into the ability of RVFV to overcome antiviral autophagy through the interaction of NSs with LC3 proteins.

## Author summary

Rift Valley fever virus (RVFV) infection can cause severe illnesses including liver disease, encephalitis, and hemorrhagic fever. Unfortunately, there are no FDA-approved vaccines or therapeutics for human use. The NSs protein is the main virulence factor of RVFV and is a potent inhibitor of the host antiviral immune responses. Autophagy has been previously shown to be antiviral in RVFV infected cells, but RVFV can suppress this antiviral pathway by a previously undetermined mechanism. In this manuscript, we demonstrate that NSs interacts with host LC3-family proteins through an LC3 interacting region (LIR) within its C-terminus. This interaction results in LC3 being retained in the nucleus and autophagy inhibition. Our studies provide mechanistic insights by which NSs subverts antiviral autophagy during RVFV infection.

## Introduction

Rift Valley fever virus (RVFV) is an arbovirus, endemic in sub-Saharan Africa, that infects both ruminants and humans [1]. Transmission occurs via mosquitos, through contact with the blood or amniotic fluid of an infected animal, or airborne via virus containing aerosols [1]. Infections of pregnant ruminants are characterized by abortion storms and fetal malformations, and spontaneous abortions occur in almost 100% of infected ruminants [2]. Ruminant disease is severe with mortality rates near 100% in young ruminants and approximately 30% in adults, which leads to severe socio-economic impacts in areas where RVFV outbreaks occur [1]. In humans, the infection is usually less severe, with the infected individual typically presenting mild symptoms such as headache, muscle pain, and general body fatigue. However, in 10% of cases more severe symptoms are observed, including hemorrhagic fever or encephalitis [3]. Studies have demonstrated that several mosquito vectors from the US and Europe such as *Cx*. *pipiens* are capable of transmitting RVFV [4], and climate change is helping to expand the geographical habitat for mosquito vectors capable of transmitting the virus [5]. Unfortunately, there are currently no FDA approved drugs or vaccines to prevent the global spread of RVFV despite its potential pathogenic and economic impact. This gap in knowledge is in large part due to a somewhat limited understanding of the biochemical mechanisms associated with the pathogenesis of RVFV. Due to its potential to cause significant social disruption and the absence of approved drugs and vaccines to prevent its global spread, RVFV is currently classified as a Category A priority pathogen by the National Institutes of Allergies and Infectious Diseases (NIAID).

As a negative-sense RNA virus (*Phlebovirus* genus, *Phenuiviridae* family, *Bunyavirales order*) [6], RVFV contains a tripartite genome composed of negative sense large (L) and medium (M) segments, as well as the ambi-sense small (S) segment. The L segment encodes the viral RNA polymerase, which is required for viral replication and transcription. The M segment encodes the glycoproteins, which are important for receptor binding and fusion as well as a nonstructural protein (NSm), that plays a key role in inhibiting apoptosis [7]. Lastly, the S segment encodes the nucleoprotein (NP) from its genomic strand, and its anti-genomic strand encodes the NSs protein [8]. NP is an RNA-binding protein which plays an essential role in packaging of the viral RNA during replication, whereas the NSs protein is the main virulence factor by virtue of its ability to form interactions with multiple cellular proteins including several subunits of the general transcription factor IIH (TFIIH), protein kinase R (PKR), and the chromatin remodeling protein SAP30 [9–16].

Upon RVFV infection, multiple signaling pathways in the host cell are induced and some of these pathways function to suppress viral replication (e.g. innate immune responses), whereas other pathways are exploited by the virus to replicate (e.g., p70 S6K signaling) [17]. One such pathway that is induced during RVFV infection is autophagy [18,19], which is the process whereby cellular material is degraded and recycled to help maintain cellular homeostasis [20,21]. A previous study found that autophagy was induced in RVFV infected cells (U2OS osteosarcoma cells), and that RVFV replication increases when the levels of key autophagy proteins (ATG13, ATG5, ATG7) were decreased via either gene deletion or targeted siRNA approaches [18]. In addition, it was determined that activation of autophagy following RVFV infection required the pattern recognition receptor Toll-7 and downstream signaling proteins TRAF6 and MyD88, indicating that a pathogen associated molecular pattern (PAMP) is triggering autophagy. Exosomes from RVFV infected cells, which contain viral RNA, NP, and viral glycoproteins, can also induce autophagy in naïve uninfected cells, which is dependent on the cytoplasmic pattern recognition receptor Retinoic acid-inducible gene I (RIG-I) [22]. Combined, these two studies suggest that following RVFV infection, pattern recognition receptor signaling stimulates autophagy to help suppress viral replication, and that autophagy is serving an antiviral role. However, a more recent study demonstrated that autophagy is activated in macrophages infected with the RVFV and that the activation is dependent on the presence of the viral NP. In this case, the activation of autophagy appears to serve a proviral function by inhibiting the host cell’s innate immune response and promoting an increase in the rate of viral replication [19]. Taken together, these results suggest that in human cells, the autophagy system can play varying roles following RVFV infection, but these roles could depend on both the cell type involved and the time after the initiation of the infection. These varying results are consistent with other studies demonstrating that activation of the host cells’ autophagy system following a viral infection can be either pro- or anti-viral [23,24].

Despite being the main virulence factor, there is very little information to date as to how the NSs protein influences autophagy following RVFV infection. Shortly after a RVFV infection, the NSs protein forms large amyloid like filaments in the nucleus [25], and these NSs filaments appear to be required for the suppression of the host immune response through the inhibition of both transcription and the interferon response. NSs suppresses transcription, in part, by forming protein-protein interactions with the p62 subunit of the TFIIH and PKR [10–16]. In both cases, the interaction with NSs leads to their degradation via the ubiquitin-proteosome system, which requires the E3-ubiquitin ligase FBXO3 for p62 degradation and the E3-ubiquitin ligase FBXW11 for PKR degradation [10–16]. In the case of the p62 subunit of TFIIH, NSs binds through interactions with a short linear motif (SLiM) known as an ΩXaV motif (where Ω is Trp or Phe, X is any amino acid, a is Asp or Glu, and V is Val) located at the very C-terminus of the protein. Interestingly, the ΩXaV motif is present in the RVFV NSs, but is not found in the NSs proteins of all other known bunyaviruses, which suggests this motif provides a unique function to the RVFV NSs [11]. In addition, the ΩXaV motif of NSs resembles a SLiM motif present in many autophagy factors commonly referred to as the LIR (LC3-Interacting Region) motif.

In humans, the six LC3-family proteins (LC3A, LC3B, LC3C, GABARAP, GABARAPL1 and GABARAPL2) are key autophagy factors that function by regulating growth of autophagic membranes, recognition of autophagic cargoes, and fusion of autophagic membranes with lysosomes [26]. LC3 proteins carry out their various functions during autophagy in part due to their ability to interact with other autophagy factors that contain LIR motifs. The core of the LIR motif consists of four amino acids that contains the canonical sequence W/F/Y-x-x-L/V/I, where x represents any amino acids (aa) [27–29] and thus the ΩXaV motif from the p62 subunit of TFIIH could also qualify as an LIR motif based on its sequence. Given the presence of the ΩXaV motif in NSs and the importance of autophagy during infections of RVFV, we searched the sequence of NSs using several databases in an attempt to identify potential LIR motifs. The searches identified four potential LIRs (referred to as NSs1-4), including one that corresponded to the C-terminal ΩXaV motif (now referred to as NSs4). Modeling studies using AlphaFold showed that NSs1-4 all had the capacity to form complexes with the different human LC3-family proteins, but analysis with FoldX suggested that NSs4 bound in the most energetically favorable manner. Biophysical characterization of NSs1-4 binding to the human LC3-family proteins using isothermal titration calorimetry (ITC) and structural characterization by X-ray crystallography supported the modeling results with AlphaFold. The results from the modeling and *in vitro* studies were further supported by cellular studies in RVFV infected cells, which showed that NSs interacts with LC3-family members and inhibits autophagy during RVFV infection, and the LIR within NSs4 was critical for this function.

## Results

### RVFV NSs contains four putative LIR motifs

**Fig 1:**
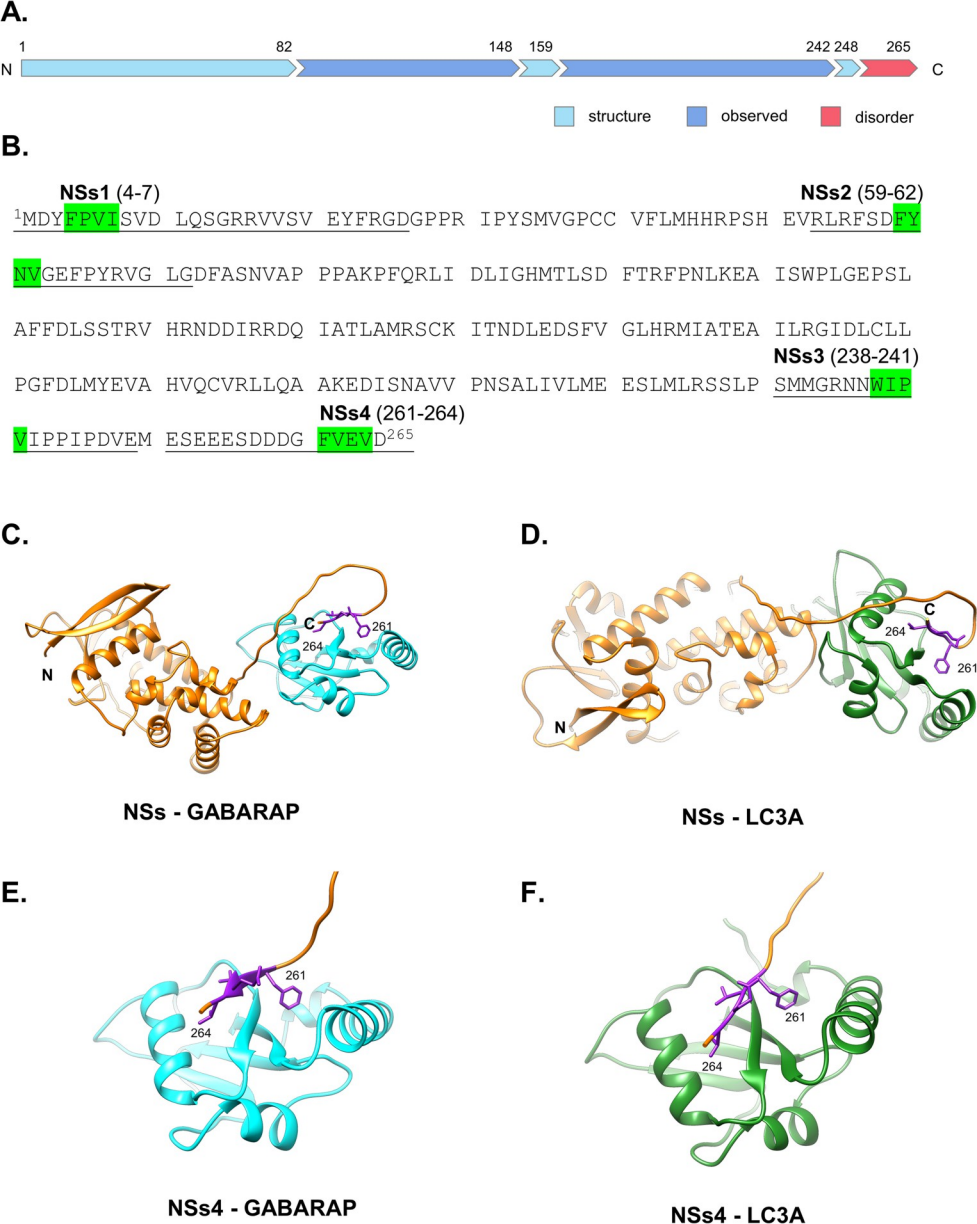
RVFV NSs interacts with LC3A and GABARAP *in silico* via LIR motifs. (A) The linear sequence of RVFV NSs (UniProt ID P21698) was annotated based on mobility and disorder analysis by MobiDB [73] and regions labeled as structured, disordered, and observed, where “observed” refers to experimentally observed residues in the protein structure (i.e., X-ray, NMR, Cryo-EM). (B) The location of potential LIR motifs predicted by ELM and iLIR were mapped onto the amino acid sequence of NSs (green shading). (C-D) Representative structural models of RVFV NSs (orange) in complex with GABARAP (cyan) and LC3A (forest green) proteins, as calculated via AlphaFold and FoldX (Materials and Methods). The C-terminal LIR motif of NSs (i.e., NSs4; purple atoms) interacts with LC3 family members in all cases. (E-F) Representative structural models of NSs4 LIR motif-containing peptide (orange) in complex with the GABARAP (cyan) and LC3A (forest green) proteins, as calculated via AlphaFold and FoldX (Materials and Methods). In the models, the NSs4 LIR motif (purple atoms) interacts with LC3A and GABARAP in a similar manner.

Given that RVFV has been shown to be regulated by autophagy [18] and that NSs is the main virulence factor for RVFV, we performed independent searches with the iLIR autophagy database [30,31] and the Eukaryotic Linear Motif (ELM) resource [32] to identify putative LIR motifs within the NSs sequence. Interestingly, these searches identified four putative LIR motifs that are located between residues 4–7 (^4^FPVI^7^), 59–62 (^59^FYNV^62^), 238–241 (^238^WIPV^241^) and 261–264 (^261^FVEV^264^) in the NSs sequence (Fig 1A–1B). Based on a structural model of the full-length NSs determined using AlphaFold, the first two putative LIRs (from here on referred to as NSs1 and NSs2, respectively) are located within a structured domain at the N-terminal end of the protein, whereas the third and fourth putative LIRs (from here on referred to as NSs3 and NSs4, respectively) are located within an intrinsically disordered region at the C-terminal end of the protein (Figs 1C and 1D and S1). The presence of four LIR motifs is interesting especially given that LIR motifs have been reported to be over-represented in viral proteins [33]. This motif is known to mediate interactions with LC3-family members, and thus is likely to enable NSs to modulate the host autophagy systems.

To assess which of the four putative LIR motifs within the NSs protein is most likely to form interactions with LC3-family proteins, structural models of the full-length NSs protein were folded in complex with each of the six members of the human LC3-family of proteins generated using AlphaFold [34,35] (Figs 1C and 1D and S1). Each complex structure was further refined by FoldX [36], which produced estimated binding energy and location. In addition, the accessible surface area (ASA) was calculated [37] to identify disordered regions in the unbound protein and quantify binding to the target LIR motif based on change in ASA when bound. Each modeling experiment yielded folded protein complexes, where the region of NSs containing the NSs4 LIR bound in the most energetically favorable manner with the six human LC3 proteins, based on biophysical parameters (Table 1). Based on the AlphaFold predicted models of the six complexes, it appears that NSs4 would be the putative LIR most likely to interact with all six of the human LC3-family proteins.

**Table 1:** Biophysical parameters of *in silico* interactions between NSs and LC3 family members.

Binding Target	Δ Multimer ASA*	Binding Energy**	Rank***
GABARAP	0.444	-28.43	7
GABARAPL1	0.437	-27.87	2
GABARAPL2	0.441	-29.89	3
LC3A	0.384	-15.46	3
LC3B	0.391	-25.16	0
LC3C	0.435	-29.27	0

*Total energy of the complex formed between NSs with the binding target LC3 family protein in kcal/mol.

**Change in accessible surface area (angstroms squared) upon binding of NSs to target LC3 protein.

***The AlphaFold seed experiment which produced the highest ranking complex out of 25 total models.

To further assess the potential of the four putative LIRs of NSs to bind LC3-family proteins, additional AlphaFold models of complexes were generated using shorter amino acid sequences corresponding to NSs1 (residues 2–16), NSs2 (residues 53–72), NSs3 (234–255) and NSs4 (247–265). For the predictions with the four peptides, GABARAP was selected as the representative protein for the GABARAP subfamily, whereas LC3A was selected as the representative protein for the LC3 subfamily. Eight models in total (4 each with LC3A and GABARAP) were generated with AlphaFold (Figs 1E and 1F, S1, and S1 Table) and the structures were analyzed using FoldX to assess the relative structural integrity binding energy, location and accessible surface area (ASA) of the models. Based on the analysis with these short amino acid sequences, NSs4 exhibited the most favorable interactions with both GABARAP and LC3A in terms of both ASA and energy, further supporting the putative role of this LIR motif of NSs in targeting LC3-family members as predicted with the full-length NSs protein. These models link the presence of discrete amino acid sequence elements in NSs with RVFV modulation of autophagy, prompting us to validate our predictions using *in vitro* and cellular systems.

### The putative LIRs in NSs interact *in vitro* with LC3-family members

To assess the ability of the four putative LIRs of NSs to bind to the LC3-family proteins *in vitro*, the six human LC3-family proteins were purified and their dissociation constants (*K*_D_) for a purified peptide corresponding to NSs4 (residues 247–265) determined using isothermal titration calorimetry (ITC) experiments. The ITC experiments (Table 2) demonstrated that the NSs4 bound with sub-micromolar affinity to all six of the human LC3-family proteins under the experimental conditions (20 mM Phosphate pH = 6.5 and 50 mM NaCl) with the exception of GABARAPL2 which bound with low micromolar affinity. The *K*_D_ values were 0.24 ± 0.05 μM for LC3A, 0.60 ± 0.04 μM for LC3B and 0.20 ± 0.06 μM for LC3C within the LC3 subfamily, whereas the *K*_D_ values were 0.43 ± 0.03 μM for GABARAP, 0.49 ± 0.030 μM for GABARAPL1 and 2.2 ± 0.1 μM for GABARAPL2 within the GABARAP subfamily. Thus, NSs4 bound with more or less similar affinities to the three members of the LC3 subfamily as well as GABARAP and GABARPL1, but with lower affinity to GABARAPL2.

**Table 2:** NSs interacts with LC3 family members *in vitro*.

	NSs4 (K_*D*_)	NSs3 (K_*D*_)	NSs2 (K_*D*_)	NSs1 (K_D_)
**LC3A**	240 ± 5 nM	n.d.*	n.d	n.d.
**LC3B**	600 ± 30 nM			
**LC3C**	200 ± 60 nM			
**GABARAP**	430 ± 30 nM	16000 ± 3.0 nM	n.d.	n.d
**GABARAPL1**	490 ± 30 nM			
**GABARAPL2**	2200 ± 100 nM			

*n.d. = no heat detected

Next, to verify that NSs4 is binding to the consensus LIR-binding site on LC3-family proteins as predicted by AlphaFold, Nuclear Magnetic Resonance (NMR) spectroscopy chemical shift perturbation experiments were performed with both LC3A and LC3B and the NSs4 peptide. ^1^H-^15^N heteronuclear single quantum coherence (^1^H-^15^N HSQC) spectra were recorded with either ^15^N-labeled LC3A (S3A Fig) or ^15^N-labeled LC3B (S3B Fig) in both the absence and presence of the NSs4 peptide. With either LC3A or LC3B, ^1^H and ^15^N chemical shift changes were observed for a number of signals following the addition of NSs4. The signals from LC3B exhibiting the most significant chemical shift changes were then mapped onto the structure of LC3B (S3C Fig) and as expected they are located in regions around hydrophobic pocket 1 (HP1) and HP2, which constitutes the consensus LIR-binding region of LC3B [30,38,39]. Next, ^1^H-^15^N HSQC spectra were performed with ^15^N-labeled NSs4 in either the absence or presence of unlabeled LC3A. Again, ^1^H and ^15^N chemical shift changes are observed for a number of signals of NSs4 following the addition of LC3A (S3D Fig). The signals exhibiting the most dramatic chemical shift changes correspond to the residues either within or immediately adjacent to the core four residues of the LIR motif present in NSs4. Taken together the NMR results support the notion that NSs4 contains an LIR motif that has the capacity to bind to LC3-family proteins in a similar manner as LIR motifs found in a number of autophagy receptors [40–43].

Since the NMR experiments indicated that NSs4 was binding in a similar manner as other LIR motifs found in factors that regulate autophagy in humans, ITC experiments were performed using peptides containing either a Ser substitution for Phe261 (NSs4 F261S) or Val264 (NSs V264S) in the LIR motif of NSs4. These two residues, but in particular the aromatic residue, are considered as the key residues for both the ΩXaV motif binding to the p62 subunit of TFIIH and for LIR motifs binding to LC3-family proteins. In addition, the F261S substitution was previously shown to result in a decrease in viral titers in human small airway epithelial cells (HSAECs) infected with RVFV [11]. Consistent with other studies showing that the aromatic residue and the hydrophobic residue in the core of the LIR motif are crucial for binding to LC3-family proteins, no heat of binding was observed between either the NSs4 F261S or the NSs4 V264S peptides and LC3A, which indicates a *K*_D_ value for binding of this complex to be >100 μM under the experimental conditions (Table 3). Together, the NMR experiments and the ITC studies with the NSs4 F261S and NSs4 V264S peptides indicate that the LIR motif in NSs4 is binding to human LC3-family proteins in a manner similar to what is observed in the models generated by AlphaFold.

**Table 3:** Phe 261 within NSs4 is important for the interaction with LC3A *in vitro*.

NSs4	LC3A (K_*D*_)
Wild-type	240 ± 50 nM
F261S	n.d.*
V264S	n.d.*

*n.d. = no heat detected

Given the results with NSs4, additional peptides containing the NSs1, NSs2 and NSs3 LIRs were purified and their *K*_D_’s for binding to GABARAP and LC3A determined using ITC studies under identical experiment conditions as used with NSs4. In the case of NSs1 and NSs2, there was no heat of binding detected with either LC3A or GABARAP, which indicates that the *K*_D_ value for binding of these putative LIR motifs to LC3-family proteins is >100 μM, under the experimental conditions (Table 2). Likewise, no heat of binding was observed between NSs3 and LC3A again suggesting a *K*_D_ value for binding of this complex is >100 μM (Table 2). In contrast, the *K*_D_ value for the binding of NSs3 to GABARAP was determined to be 16 ± 3.0 μM (Table 2). Taken together, the *in vitro* ITC studies and NMR experiments are consistent with the AlphaFold results with the full-length NSs protein, which suggests that NSs4 is the most likely of the four putative LIR motifs in NSs to interact with the human LC3-family proteins during a RVFV infection.

### Structures of NSs4 and NSs3 in complex with LC3-family proteins

To structurally define the interactions between NSs4 and LC3-family proteins at the atomic level, attempts were made to generate co-crystals of NSs4 in complex with the six human LC3 proteins as well as for the complex between NSs3 and GABARAP. After extensive screening of different crystallization conditions, no co-crystals were obtained with NSs4 and any of the six human LC3 proteins. However, crystals were obtained by fusing NSs4 to the N-terminus of either GABARAP or LC3A, a method commonly used to examine the structures of LIR motifs with LC3-family protein [44–46]. The crystals obtained with the NSs4-LC3A and the NSs4-GABARAP fusion proteins diffracted to 2.4Å and 2.0Å resolution, respectively (S2 Table). In contrast, we obtained co-crystals of a complex between NSs3 and GABARAP that diffracted to 1.9Å resolution (S2 Table).

**Fig 2:**
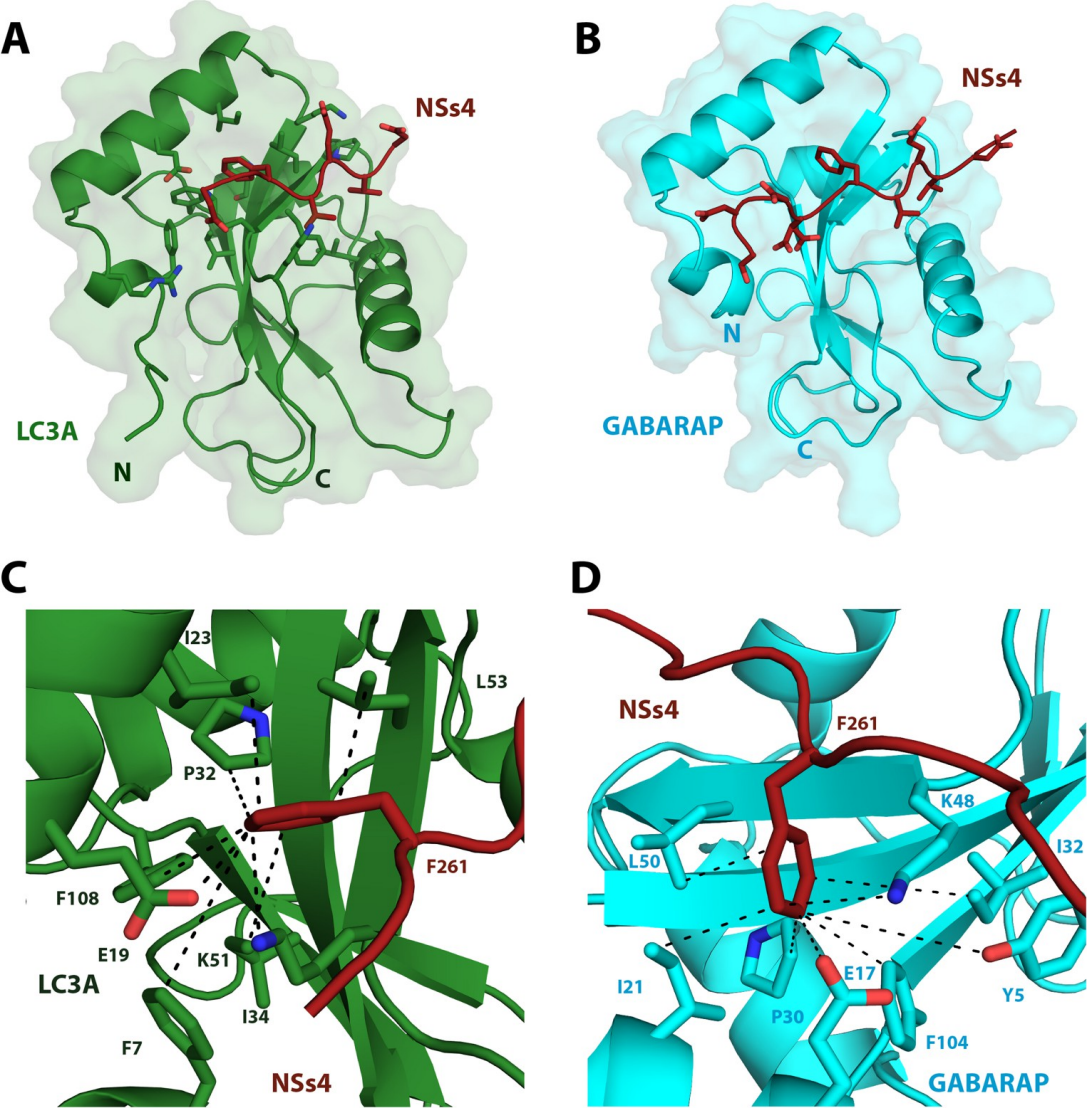
Crystal structures of NSs4 in complex with LC3A and GABARAP. (A) Cartoon representation of the crystal structure of the NSs4-LC3A complex highlighting the side chains of NSs4 (firebrick red) when bound to LC3A (forest green). (B) Cartoon representation of the crystal structure of the NSs4-GABARAP complex highlighting the side chains of NSs4 (firebrick red) when complexed with GABARAP (cyan). (C) Close-up and metrics (in Å) of the NSs4-LC3A complex highlighting the side chains of amino acids from HP1 of LC3A (forest green) that make either hydrophobic (F7, I23, P32, I34, L53, F108), anion-π (E19) or cation-π (K51) interactions with the side chain of F261 of NSs4 (firebrick red) at the binding interface. (D) Close-up and metrics (in Å) of the NSs4-GABARAP complex highlighting the side chains of amino acids from HP1 of GABARAP (cyan) that make either hydrophobic (Y5, I21, P30, I32, L50, F104), anion-π (E17) or cation-π (K48) interactions with the side chain of F261 of NSs4 (firebrick red) at the binding interface. The dashed lines (black) in panels C-D correspond to the distance measurements given in the text for the key interactions at the interfaces of the complexes.

In complex with NSs4, both LC3A and GABARAP adopt the canonical Atg8 fold (Fig 2A and 2B), similar to what has been observed in previously reported structures of their free forms as well as in other structures of fusion proteins with LIR motifs. Likewise, NSs4 bound in a linear conformation to both LC3A and GABARARP in a similar manner to what has been observed for a typical LIR motif in complex with an LC3 protein (Fig 2A and 2B). In both complexes, the predominant interactions at the binding interfaces involve the side chains of F261 and V264 of NSs4, which are the residues located in the first and fourth position of the core LIR motif (Fig 2C and 2D). These residues insert into HP1 and HP2 on the surfaces of LC3A and GABARAP respectively, which is consistent with most other previously determined structures of LIR motifs in complex with LC3-family proteins [27–29]. In complex with LC3A, the aromatic ring of F261 of NSs4 forms hydrophobic interactions with LC3A involving the side chains of F7 (6.3Å) I23 (4.2Å), P32 (4.2Å), I34 (5.3Å), L53 (4.1Å) and F108 (4.1Å) as well as anion-π and cation-π interaction with E19 (3.3Å) and K51 (4.2Å) in HP1 (Fig 2C). In a similar manner, the aromatic ring of F261 of NSs4 forms the corresponding hydrophobic interactions with the side chains of Y5 (6.3Å), I21 (4.1Å), P30 (3.7Å), I32 (5.8Å), L50 (4.8Å) and F104 (3.9Å) as well as anion-π and cation-π interaction with E17 (4Å) and K48 (4.1Å) in HP1 of GABARAP (Fig 2D). In addition to the interactions involving F261, the side chain of V264 of NSs4 forms hydrophobic contacts with the side chain of F52 (5.3Å), V54 (4.3Å), P55 (4.1Å), V58 (6.8Å), L63 (6.8Å) and I66 (3.9Å) in HP2 of LC3A (S4A Fig) whereas, the corresponding hydrophobic interactions in HP2 of GABARAP occur between the side chain of V264 of NSs4 and the side chains of Y49 (4Å), V51 (4.1Å), P52 (4Å), L55 (3.7Å), F60 (4.5Å) and L63 (3.4Å) (S4B Fig). The binding interfaces with the LC3 proteins are also stabilized by additional interactions, within and after the core four residues of the LIR motif of NSs4. Additional hydrophobic interactions are observed between the side chains of V262-K49 (3.6 Å) and V262-F52 (3.8Å) of NSs4 and LC3A, respectively which is comparable with the corresponding hydrophobic interactions between V262-K46 (3.9Å) and V262-Y49 (3.9Å) of NSs4 and GABARAP, respectively (S4C–S4D Fig). Interestingly, in the NSs4-LC3A complex, an anion-π interaction is formed between E263-H27 (3.4Å) and electrostatic interactions are formed between D259-R10 (4.9Å) as well as between D265-K30 (5.1Å) of NSs4 and LC3A, respectively (S4C Fig). Similarly, in the NSs4-GABARAP complex, D259 of NSs4 forms two possible interactions, either an anion-π interaction with H8 (2.9Å) or an electrostatic interaction with K48 (5.7Å) of GABARAP as well as an electrostatic interaction between E263 of NSs4 and R28 (4.7Å) of GABARAP (S4D Fig). In total, the binding interface of the NSs4-LC3A complex involves F7, R10, E19, I23, H27, K30, P32, I34, K49, K51, F52, L53, V54, P55,P58, L63, I66 and F108 of LC3A and D259, F261, V262, E263, V264 and D265 of the NSs4 whereas the binding interface of the NSs4-GABARAP complex involves Y5, H8, E17, I21, R28, P30, I32, K46, K48, Y49, L50, Y49, V51, P52, L55, F60, L63 and F104 of GABARP and D259, F261, V262, E263 and V264 of NSs4.

In the co-crystal structure of the NSs3-GABARAP complex (S5A Fig), the aromatic ring of W238 of NSs3 forms hydrophobic interactions with the side chains of Y5 (6.3Å), I21 (3.7Å), P30 (4.5Å), I32 (5.5Å), L50 (4.7Å) and F104 (3.3Å) as well as anion-π and cation-π interaction with E17 (3.8Å) and K48 (4.4Å) in HP1 of GABARAP (S5B Fig). In HP2, the side chain of V241 from NSs3 forms hydrophobic contacts with Y49 (3.9Å), V51 (3.8Å), P52 (3.3Å), L55 (4.5Å), F60 (4.6Å) and L63 (3.5Å) of GABARAP (S5C Fig). In addition, there are hydrophobic interactions from I239 of NSs3 to K46 (3.6Å) and Y49 (3.9Å) of GABARAP, a hydrogen bond interaction from N236 of NSs3 to K46 (4Å) from GABARAP and a hydrophobic interaction from V242 and the aliphatic portion of the side chain of R28 (3.9Å) from GABARAP (S5D Fig). Lastly, the side chains of Pro240, Pro243 and Pro244 of NSs3 form hydrophobic contacts with the side chains of L50 (4.2Å), L55 (4.2Å) and Q59 (3.9Å) of GABARAP, respectively (S5D Fig). In total, the binding interface of the NSs3-GABARAP complex involves residues Y5, E17, I21, R28, P30, I32, K46, K48, Y49, L50, V51, P52, L55, Q59, F60, L63 and F104 of GABARP and residues N236, I239, P240, V241, P243, P244 of NSs3.

### NSs interacts with LC3 family members in RVFV infected cells

**Fig 3:**
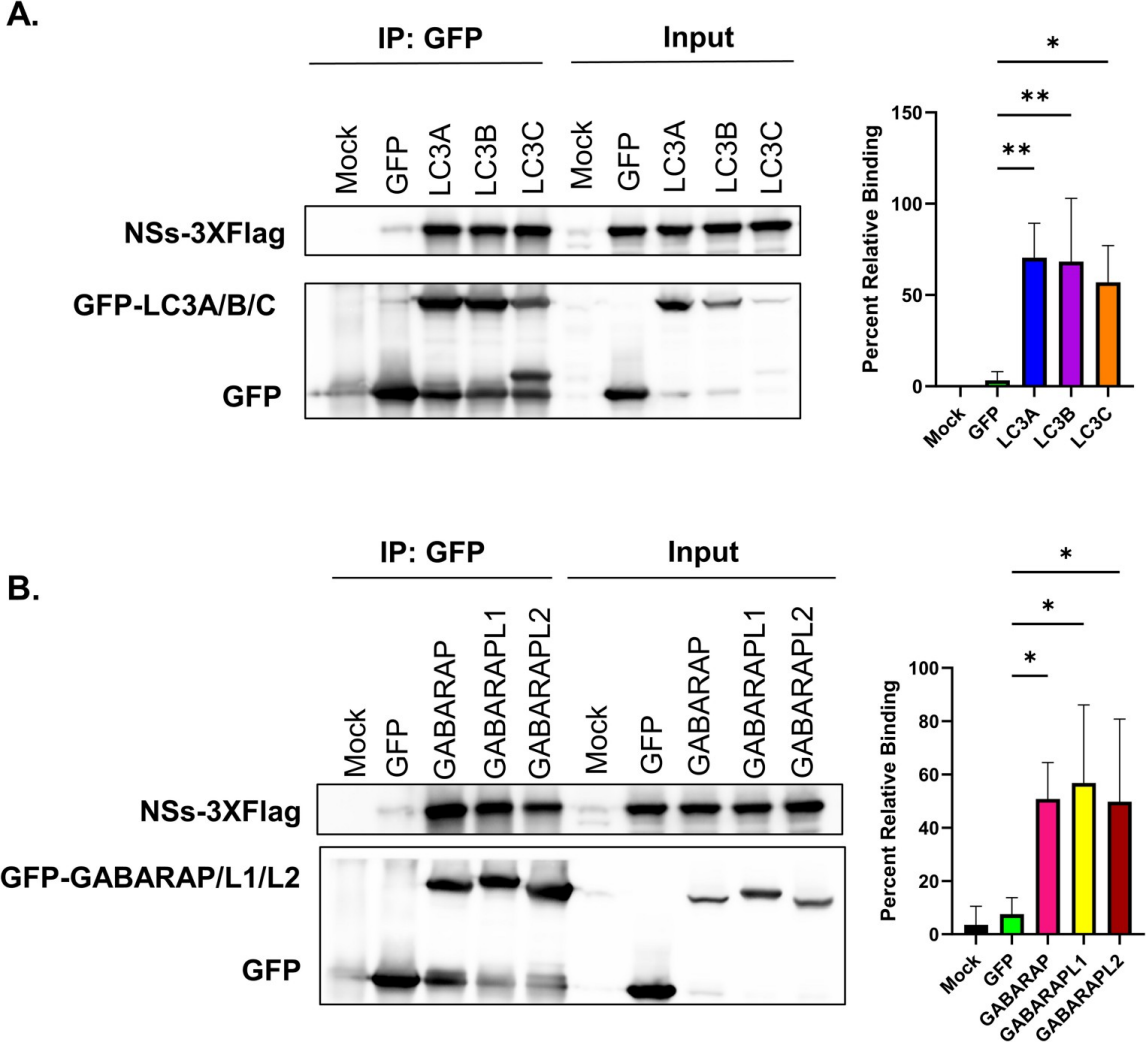
NSs interacts with LC3 family members in RVFV-infected cells. (A) BSR-T7/5 cells were transfected with media alone (mock), GFP-tagged plasmid alone (GFP), or GFP-tagged LC3A, LC3B, or LC3C plasmids and 24 h post-transfection samples were infected with MOI 0.1 rMP-12 NSs-3XFlag. Samples were collected 24 h after infection and subjected to co-immunoprecipitation with an anti-GFP specific antibody followed by western blot analysis with FLAG and GFP antibodies. (B) BSR-T7/5 cells were transfected with media alone (mock), GFP-tagged plasmid alone (GFP), or GFP-tagged GABARAP, GABARAPL1, or GABRAPL2 plasmids and 24 h post-transfection samples were infected with MOI 0.1 rMP-12 NSs-3XFlag. Samples were collected 24 h after infection and subjected to co-immunoprecipitation with an anti-GFP specific antibody followed by western blot analysis with the Flag or GFP antibodies. Quantification of band intensity for each condition is shown on the right from four biological replicates. Statistical analysis was conducted using a one-way ANOVA and post-hoc sidak’s test with * = P ≤ 0.05 and ** = P ≤ 0.01.

To verify that LC3-family proteins interact with NSs in RVFV-infected cells, BSR-T7/5 cells were transfected with plasmids expressing GFP-tagged fusions proteins of the six human LC3 family members under the control of a T7 polymerase promoter. Although RVFV NSs potently suppresses host transcription [47], BSR-T7/5 cells stably express T7 polymerase, which enables the exogenous expression of GFP-LC3 family members in the presence of NSs. Mock-transfected and GFP only transfected cells were included as controls. Twenty-four hours following transfection, cells were infected with RVFV rMP-12 NSs-3XFlag, in which a triple tandem flag is incorporated at the C-terminus of the NSs protein. Twenty-four hours post infection (hpi), cells were lysed and subjected to co-immunoprecipitation with an anti-GFP antibody, and then western blot analysis was performed using anti-GFP and anti-Flag antibodies. The analysis of the co-immunoprecipitation samples indicated that NSs has the capacity to interact with LC3A, LC3B, LC3C (Fig 3A) as well as GABARAP, GABARAPL1, and GABARAPL2 (Fig 3B). These data are consistent with the ITC results indicating that NSs can interact with all six human LC3-family members.

### NSs F261S has a significantly reduced interaction with LC3A and LC3B

**Fig 4:**
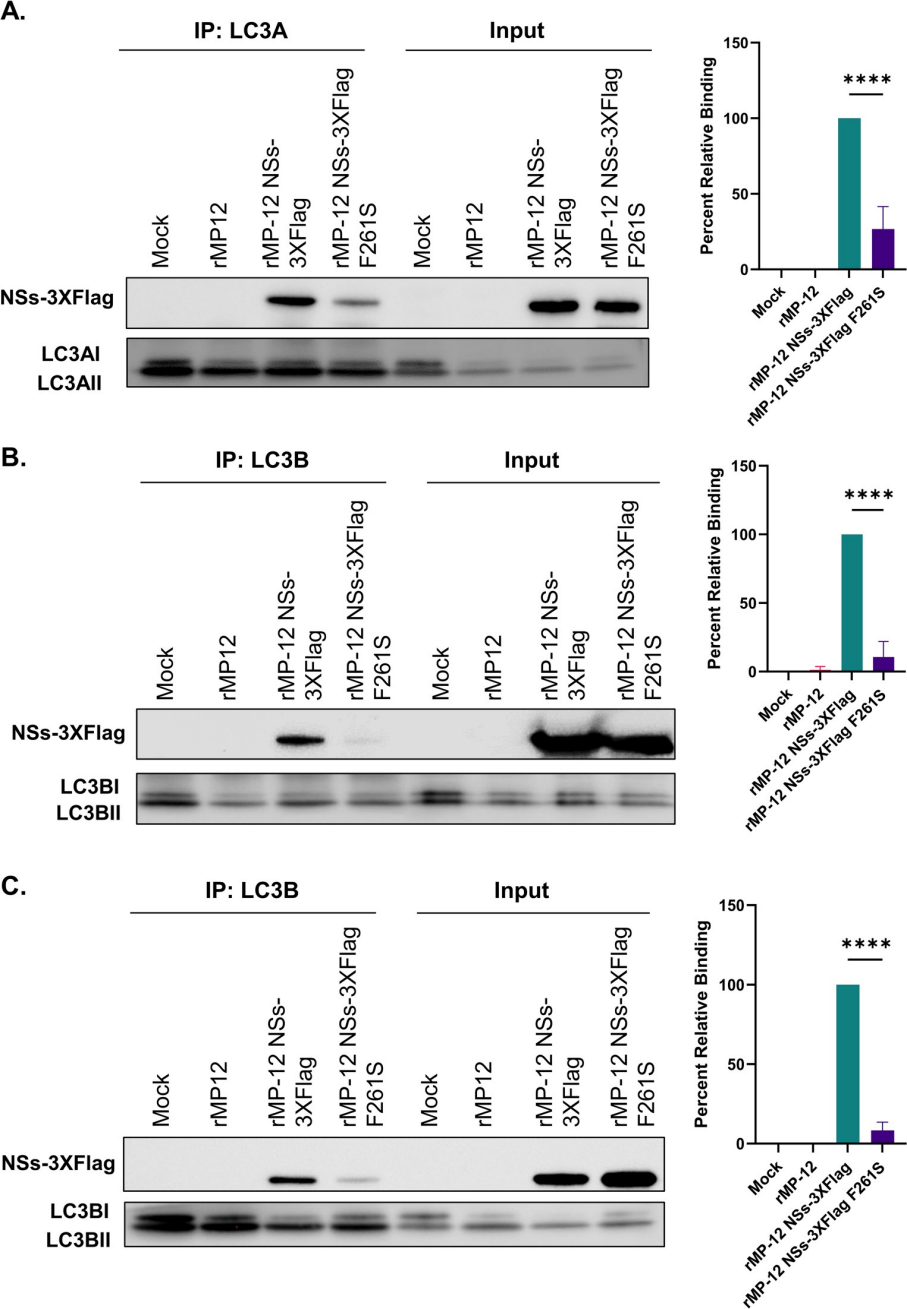
F261S substitution within the NSs4 LIR significantly reduces the interaction of NSs and LC3. HSAEC cells or Vero cells were infected at an MOI of 3 with (r)MP12, rMP-12 NSs-3XFlag, rMP-12 NSs-3XFlag F261S, or media alone (mock). Samples were collected 24 hpi and subjected to co-immunoprecipitation with either an LC3A (panel A) or an LC3B (panels B and C) specific antibody followed by western blot analysis with the appropriate antibodies. Panels A and B displays results from HSAECs and panel C shows results from Vero cells. Quantification of band intensity for each condition is shown on the right from three biological replicates. rMP-12 NSs-3XFlag was set to 100% relative binding. Statistical analysis was conducted using a one-way ANOVA and post-hoc sidak’s test with *** = P ≤ 0.001 and **** = P ≤0.0001.

To determine whether the F261S substitution in the LIR motif of NSs4 (^261^FVEV^264^) also inhibits NSs binding to LC3-family proteins in RVFV-infected cells, rMP-12 NSs-3XFlag or the NSs-Flag with the F261S substitution within NSs4 (rMP-12 NSs-3XFlag F261S) were utilized [11]. The aromatic amino acid within the LIR motif (F261 for NSs) has been shown in numerous other studies to be the key amino acid required for the binding of LIR motifs to LC3-family proteins [40]; therefore, we focused on the importance of this amino acid in our cell based assays. Cells were infected with rMP-12 NSs-3XFlag or rMP-12 NSs-3XFlag F261S and mock infected (media only) or parental (r)MP-12 infected cells were also included as negative controls. Twenty-four hpi cells were lysed and co-immunoprecipitation was performed using either anti-LC3A or anti-LC3B antibodies and western blot analysis was conducted with an anti-Flag antibody as well as with either anti-LC3A or anti-LC3B antibodies. The analysis of the co-immunoprecipitation reactions confirmed that NSs has the capacity to interact with LC3A or LC3B at endogenous levels (Fig 4) in RVFV infected HSAECs and Vero cells (Fig 4A–4B and 4C, respectively). It should be noted that LC3 proteins are present in two forms in cells, LC3-I and LC3-II, with LC3-II being the lipidated form that becomes associated with autophagosomal membranes [48]. In contrast to what was observed with the wild-type NSs, a statistically significant loss in binding to both LC3A (Fig 4A) and LC3B (Fig 4B and 4C) was observed in the rMP-12 NSs-3XFlag F261S infected cells as compared to the rMP-12 NSs-3XFlag infected cells. These results confirm that NSs4 is a functional LIR within NSs, and that the aromatic residue F261 within the LIR motif plays a key role in the interaction between NSs and either LC3A or LC3B in agreement with the ITC results.

### LC3 is predominantly nuclear in RVFV infected cells and interacts with NSs in the nucleus

**Fig 5:**
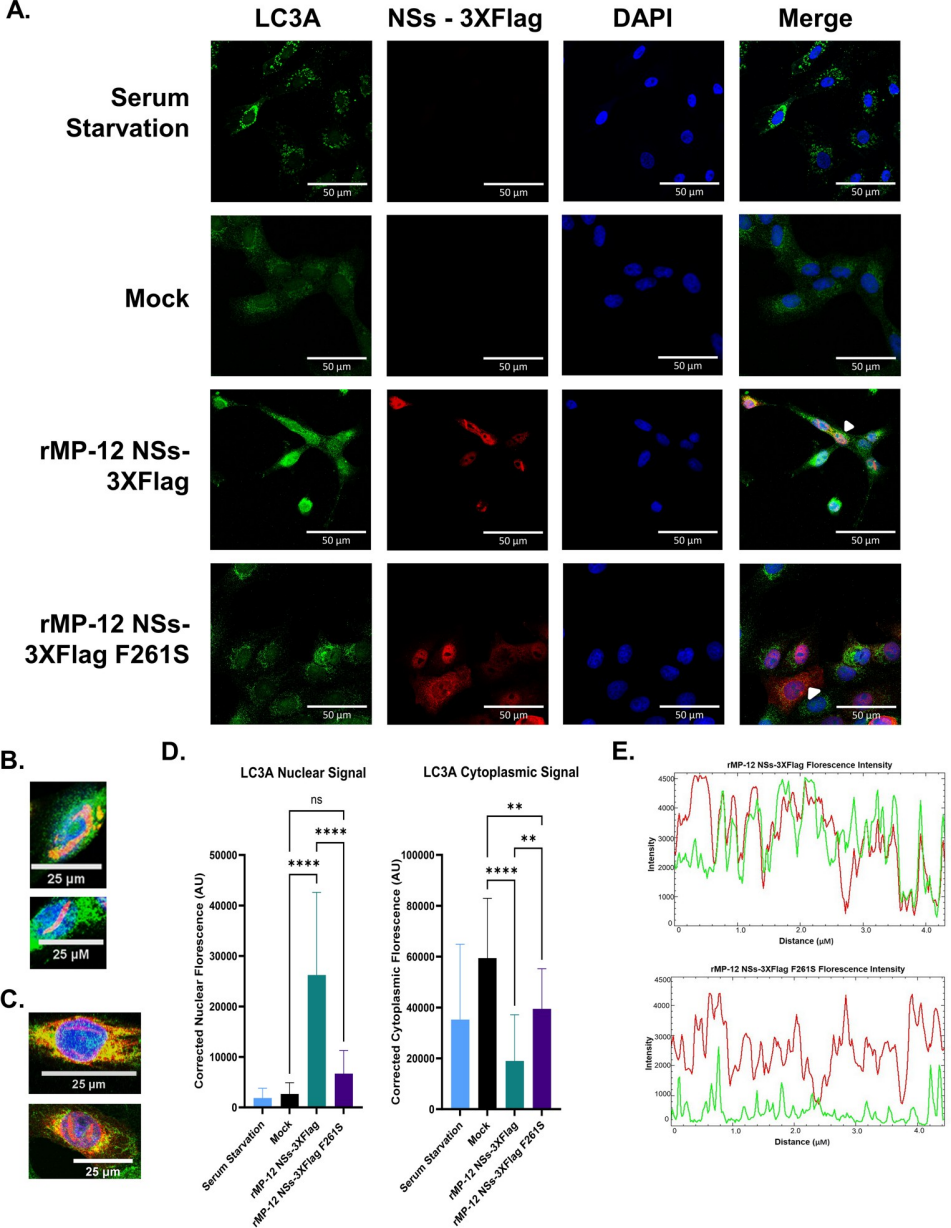
LC3A is predominantly nuclear in RVFV infected cells and colocalizes with NSs. (A) Vero cells were grown on coverslips and mock infected (media alone), infected with rMP-12 NSs-3XFlag, or infected with rMP-12 NSs-3XFlag F261S. Vero cells treated with serum starvation media is included as a positive control. The cells were stained for LC3A, NSs-Flag, and DAPI (nuclear), followed by confocal microscopy imaging. (B-C) Colocalization of LC3A and NSs was found in filaments and perinuclear regions. (D) Nuclear and cytoplasmic corrected fluorescence LC3A levels were quantified using FIJI ImageJ Software and statistically analyzed using one-way ANOVA with the GraphPad Prism Software. Twenty-five cells across three biological replicates per condition were utilized in the quantification. Statistical analysis was conducted using a one-way ANOVA and post-hoc sidak’s test with ns = not significant (P > 0.05), ** = P ≤ 0.01, and **** = P ≤0.0001. (E) Fluorescence intensity profiles of either a rMP-12 NSs-3XFlag or rMP-12 NSs-3XFlag F261S infected cell. The white arrows in panel A indicated the cells selected for analysis.

Both RVFV NSs and the human LC3-family proteins have been shown to localize in both the nucleus and the cytoplasm of cells [47,49]. Therefore, confocal microscopy experiments were performed to verify that NSs co-localizes with LC3A in cells as well as to determine in which cellular compartment(s) they co-localize. To perform the co-localization studies, Vero cells were infected with rMP-12 NSs-3XFlag, rMP-12 NSs-3XFlag F261S, or a mock infected negative control. As a positive control, uninfected cells were serum starved to induce autophagy, which results in the formation of distinct LC3A foci in the cytoplasm (Fig 5A). Next, we examined both uninfected and RVFV infected Vero cells that were not subjected to serum starvation. In the uninfected non-starved cells, LC3A was primarily localized in a diffuse manner in the cytoplasm, whereas LC3A displayed a more predominant nuclear distribution in non-starved rMP-12 NSs-3XFlag cells as compared to mock infected cells (Fig 5A and 5D). In contrast, non-starved cells infected with rMP-12 NSs-3XFlag F261S had significantly less nuclear LC3A and an increase in cytoplasmic LC3A when compared to the rMP-12 NSs-3XFlag infected cells (Fig 5A and 5D). NSs F261S distrubtion was found to be more cytoplasmic with reduced filament formation, compared to wildtype NSs, consistent our previous results [11]. These results indicate that F261 of NSs plays a key role in promoting the nuclear localization of LC3A in RVFV infected cells.

The confocal microscopy results demonstrated that there was a nuclear (Fig 5A), filamentous (Fig 5B), and perinuclear (Fig 5C) co-localization of NSs and LC3A in RVFV infected Vero cells. Based on the analysis of the images (25 cells/treatment), the Pearson’s correlation coefficient for the co-localization of LC3A and NSs in infected cells was determined to be 0.65. In contrast, co-localization of LC3A and NSs-F261S had a Pearson’s correlation coefficient of 0.01. In addition, a fluorescence intensity profile of a rMP-12 NSs-3XFlag infected cell demonstrated a clear overlap in the red (LC3A) and green (NSs) fluorescence spectra, while minimal overlap was observed during rMP-12 NSs-3XFlag F261S infection (Fig 5E). NSs and LC3A co-localization was also observed in HSAECs (S6 Fig). Taken together, these results indicate that NSs and LC3A co-localize predominantly in the nucleus and that this co-localization is highly dependent on F261 within the NSs4 LIR motif.

**Fig 6:**
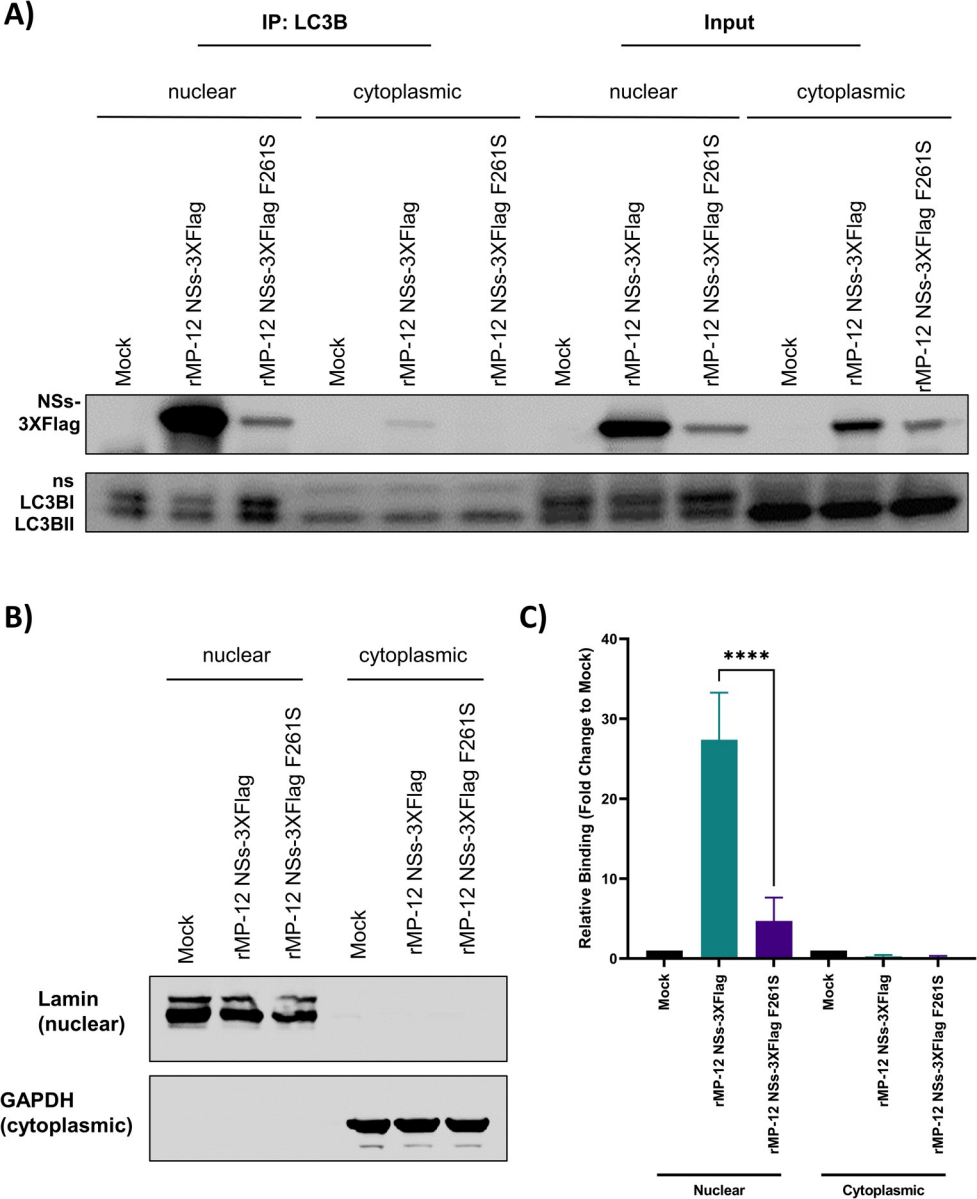
NSs interacts with LC3B in the nucleus. (A) HSAECs were infected with rMP-12 NSs-3XFlag (MOI 3), rMP-12 NSs-3XFlag F261S (MOI 3), or media alone (mock) and 24 hpi nuclear and cytoplasmic protein extracts were produced and immunoprecipitation performed with an anti-LC3B antibody. Western blot analysis was performed with anti-Flag and anti-LC3B antibodies. (B) Cytoplasmic and nuclear fraction purity was assessed via western blot analysis using anti-GAPDH and anti-Lamin A/C antibodies. (C) Quantification of band intensity for each condition is shown on the right from five biological replicates. Mock infected samples were set to a fold change of 1. Statistical analysis was conducted using a one-way ANOVA and post-hoc sidak’s test with *** = P ≤ 0.001.

To confirm that the interaction between NSs and LC3-family proteins occurs predomininantly in the nucleus, we isolated cytoplasmic and nuclear fractions from mock, rMP-12 NSs-3XFlag, and rMP-12 NSs-3XFlag F261S infected cells. Co-immunoprecipitations were performed with each cellular fraction and the results showed NSs and LC3B predominantly interacted in the nucleus (Fig 6A and 6C). In agreement with the results presented in Fig 4, the interaction between NSs and LC3B was significantly reduced in rMP-12 NSs-3XFlag F261S infected cells. Western blot analysis of lamin and GAPDH confirmed that the nuclear and cytoplasmic fractions were pure, respectively (Fig 6B). In addition, LC3B-II, which is the lipidated form of LC3B associated with the induction of autophagy, was found predominantly located in the cytoplasm (Fig 6B), wherase the nonlipated form of LC3B, LC3B-I was primarily found in the nucleus. Collectively, these results indicate that the interaction between NSs and the LC3 proteins occurs predominantly in the nucleus of RVFV infected cells and suggests that NSs interacts with the non-lipidated LC3.

### NSs suppresses autophagy through the F261 within the NSs4 LIR motif

**Fig 7:**
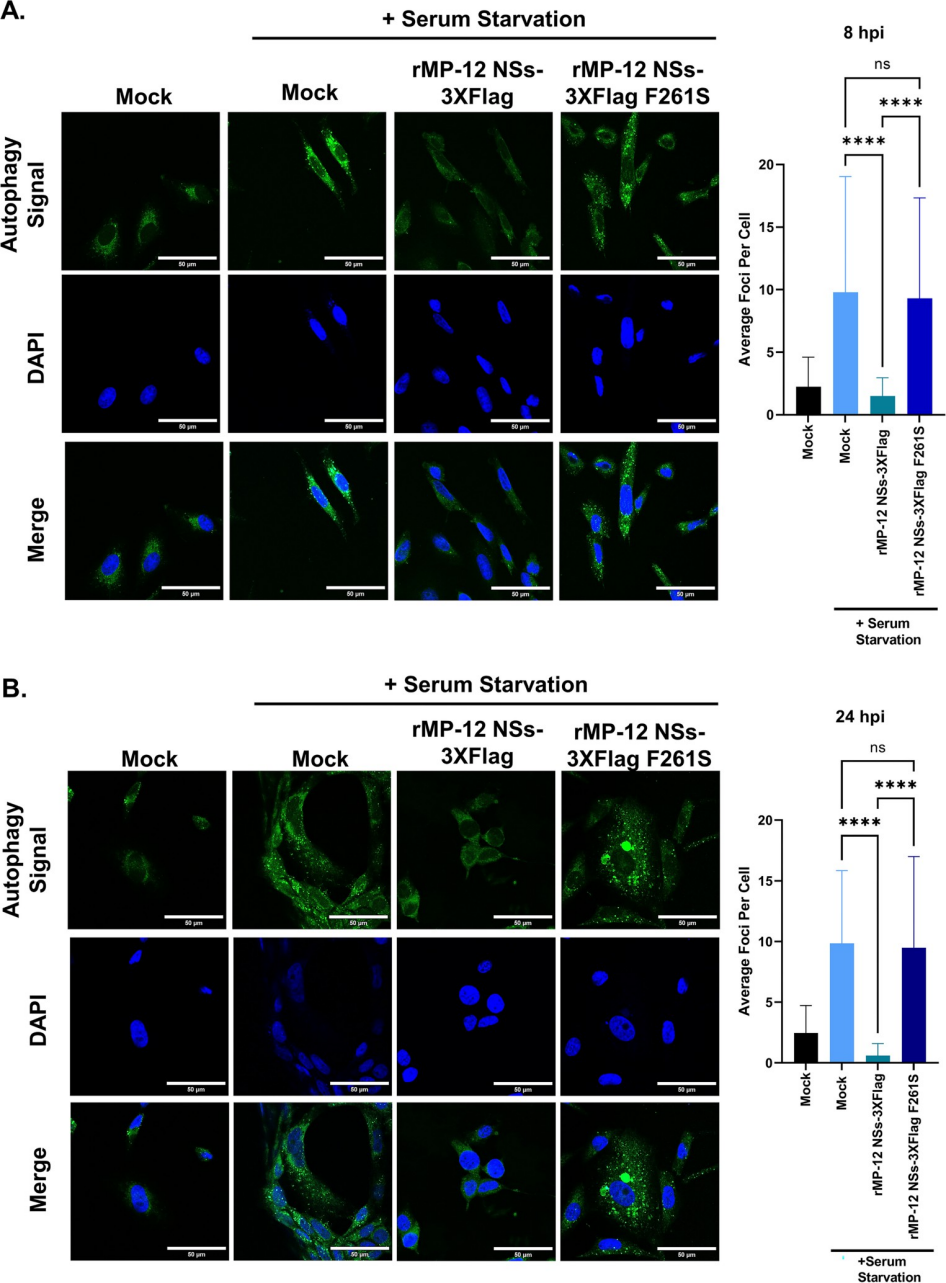
NSs suppresses autophagy through the F261 within the NSs4 LIR motif in Vero cells. Cells were analyzed using the Abcam Autophagy Detection Kit Protocol, followed by confocal microscopy imaging. Green signal indicates autophagic vesicle formation. DAPI (blue) is the nuclear marker. Uninfected cells in complete media serve as negative controls. Mock infected, rMP-12 NSs-3XFlag (MOI 3), or rMP-12 NSs-3XFlag F261S (MOI 3) infected cells were subjected to serum starvation 4 hours prior to collection. Panel A is 8 hpi and Panel B is 24 hpi. The average foci per cell (50 cells/condition) is shown on the right of each panel. Statistical analysis was conducted using a one-way ANOVA and post-hoc sidak’s test with ns = not significant (P > 0.05) and **** = P ≤0.0001.

To assess the impact of the F261 on the ability of NSs to inhibit autophagy during RVFV infection, a 488nm excitable green florescent dye that selectively incorporates into autophagic vacuoles was used in Vero cells that were mock infected, infected with rMP-12 NSs-3XFlag, or infected with rMP-12 NSs-3XFlag F261S. To initiate autophagy, cells were subjected to serum starvation for 4 h prior to collection at either 8 hpi or 24 hpi to evaluate the impact of the F261S substitution on autophagy induction. Mock infected cells that were not serum starved were included for both the 8 hpi and 24 hpi points to serve as negative controls. As expected, very few cytoplasmic autophagic foci were observed in the mock infected cells at either 8 hpi or 24 hpi (Fig 7A and 7B). In contrast, the mock infected cells subjected to serum starvation displayed numerous autophagic foci per cell at both 8hpi and 24 hpi (Fig 7A and 7B). However, significantly fewer foci per cell were observed in the infected cells with the rMP-12 NSs-3XFlag at both 8 hpi and 24 hpi, indicating that autophagy is suppressed following RVFV infection. In contrast, infected cells with the rMP-12 NSs-3XFlag F261S had statistically more foci per cell on average when compared to the infected cells with the rMP-12 NSs-3XFlag at both 8 hpi and 24 hpi (Fig 7A and 7B).

**Fig 8:**
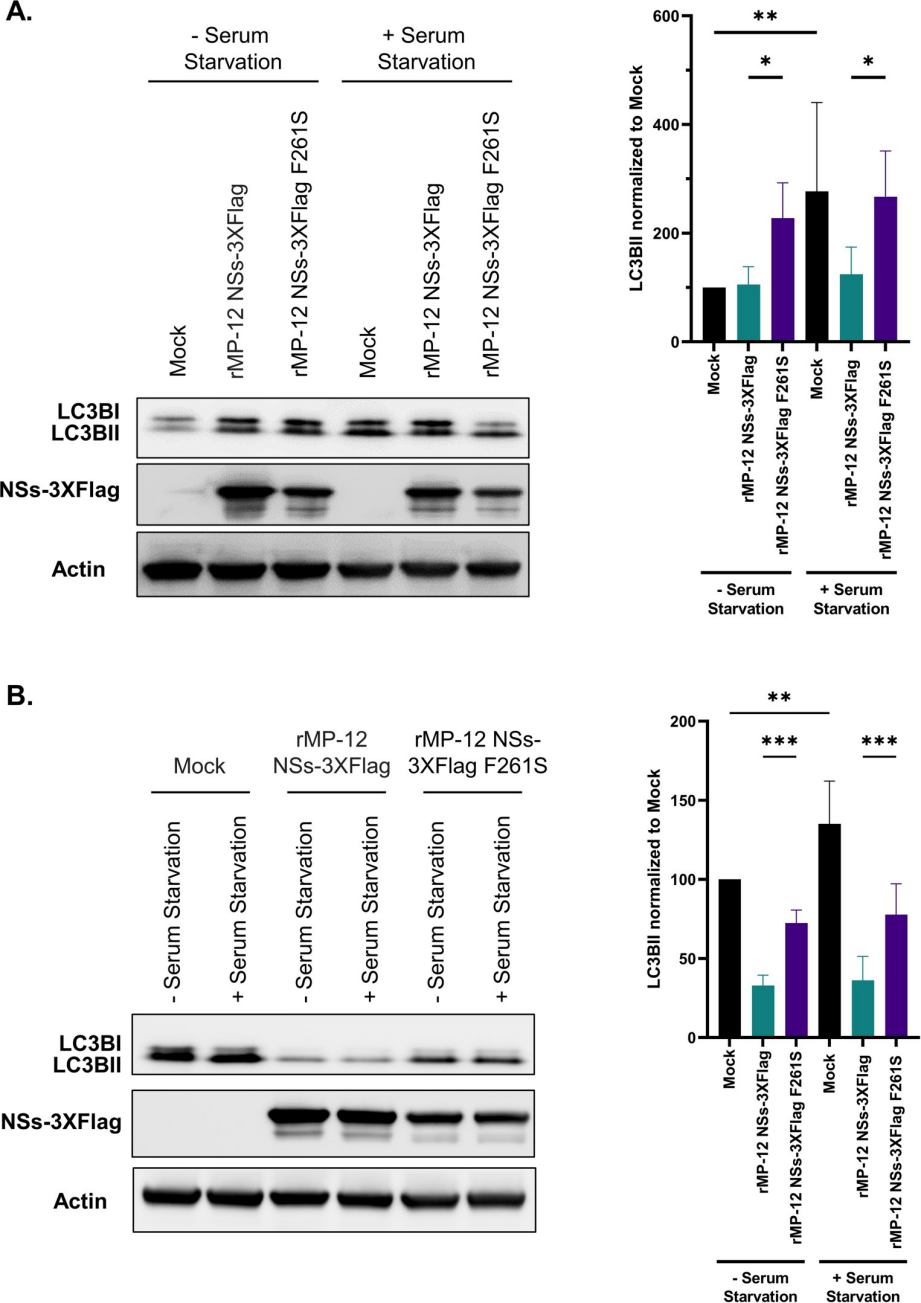
NSs reduces LC3BII through the F261 within the NSs4 LIR motif. Vero cells were infected with rMP-12 NSs-3XFlag (MOI 3) or rMP-12 NSs-3XFlag F261S (MOI 3). Uninfected (Mock) cells served as a control. In addition, mock infected, rMP-12 NSs-3XFlag, or rMP-12 NSs-3XFlag F261S infected cells were subjected to serum starvation 4 h prior to collection. Panel A is 8 hpi and Panel B is 24 hpi. Cell lysates were collected and analyzed for LC3B, NSs-3XFlag, and actin expression via western blot analysis. Quantification of LC3BII band intensity for each condition is shown on the right from six biological replicates. LC3II band intensity was normalized to actin, then mock infected cells without serum starvation were set to 100%, and all other samples normalized to this. Statistical analysis was conducted using a one-way ANOVA and post-hoc sidak’s test with ** = P ≤0.01 and **** = P ≤0.0001.

Alterations in autophagy were also assessed by analyzing changes in the lipidated LC3BII levels in cells with and without serum starvation. Measurement of LC3II levels are recognized as a reliable measurement of autophagy, as opposed to the ratio of LC3II to LC3I which can be impacted by antibodies having greater affinity for LC3II [50]. LC3BII levels were increased in mock infected cells subjected to serum starvation at both 8 and 24 hpi (Fig 8A and 8B). rMP12 NSs-3XFlag infected cells had reduced LC3BII levels compared to rMP-12 NSs-3XFlag F261S infected cells at both 8 and 24 hpi with and without serum starvation. A potentially confounding factor is that there is less overall LC3B present in both rMP-12 NSs-3XFlag and rMP-12 NSs-3XFlag F261S infected cells at 24 hpi, which is consistent with the ability of RVFV to shutdown host transcription and translation [51–53]. However, such a decrease is not observed at 8 hpi, which highlights the ability of NSs to reduce LC3BII through the F261 within the NSs4 LIR motif. Collectively, these data indicate that NSs suppresses autophagy and that the aromatic residue F261 in the NSs4 LIR motif plays a key role in this suppression.

### The lack of an interferon response can rescue the attenuated F261S phenotype

**Fig 9:**
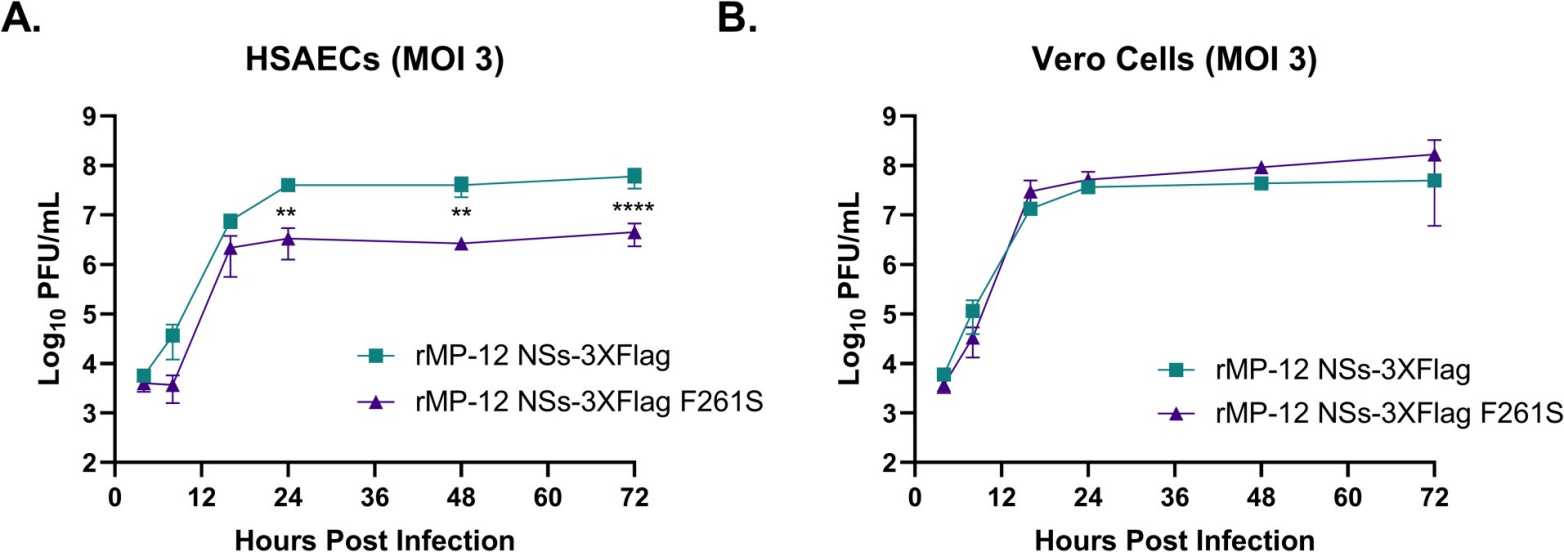
rMP-12 NSs-3XFlag F261S has reduced replication capability in HSAECs, but not Vero cells. HSAECs (panel A) or Vero cells (panel B) were infected with rMP-12 NSs-3XFlag or rMP-12 NSs-3XFlag F261S at an MOI of 3. Supernatants were collected at 4, 8, 16, 24, 48, and 72 h post infection and viral titers determined by plaque assay. Data presented are the averages of three biological replicates. Statistical analysis was conducted using a one-way ANOVA and post-hoc sidak’s test with ** = P ≤0.01 and **** = P ≤0.0001.

There is significant crosstalk between autophagy and the interferon response [23,54]. Autophagy has been shown to facilitate viral recognition via pattern recognition receptors, which contributes to interferon induction [55–57]. Moreover, NSs F261S is deficient in TFIIF p62 binding and degradation, which contributes to interferon suppression in RVFV infected cells [10,12,53]. Given this, experiments were performed to determine if loss of a functional interferon response would impact rMP-12 NSs-3XFlag F261S replication kinetics. Vero cells (interferon deficient) and HSAECs (interferon competent) were infected with rMP-12 NSs-3XFlag and rMP-12 NSs-3XFlag F261S and viral titers determined up to 72 hpi. In agreement with our previous results [11], rMP-12 NSs-3XFlag F261S replicated to lower levels compared to rMP-12 NSs-3XFlag in HSAECs, with statistical differences observed at 24, 48, and 72 hpi (Fig 9A). In contrast, rMP-12 NSs-3XFlag and rMP-12 NSs-3XFlag F261S replicated similiarly in Vero cells (Fig 9B). These data indicate that the lack of an interferon response can rescue the attenuated F261S phenotype.

## Discussion

In humans, serious infections with RVFV can progress to hemorrhagic fever, encephalitis, neurological disorders, ocular disease, and organ failure and there is an ever increasing opportunity for the emergence of this potentially lethal virus in the U.S and Europe as competent mosquito vectors have already been identified [3]. Identifying the interactions of the NSs protein, the main virulence factor of RVFV, with host proteins is crucial to helping understand the progression of human infections as well as helping with the development of potential vaccines and therapeutic agents against the virus [12]. In this manuscript, we have characterized at the atomic level interactions between the six human LC3 proteins and an LIR motif present in NSs as well as investigated the impact that NSs has on regulating cellular autophagy during infection with the RVFV. Utilizing bioinformatics and AlphaFold analysis, we identified four potential LIR motifs (NSs1-4) in the NSs protein and demonstrated that NSs had the capacity to complex with the six human LC3 proteins through the LIR motif located at the very C-terminus of the protein (NSs4). Using a combination of structural, biophysical and functional studies, we verified that F261 within the NSs4 LIR motif was crucial for the interaction with the LC3 proteins and for the suppression of autophagy in cells infected with the RVFV.

Viruses have limited coding capacity and therefore viral proteins are often required to interact with a multitude of host proteins to perform various functions to ensure the survival of the virus as well as its ability to replicate within the host. The multifunctional nature of viral proteins is often mediated using SLiMs due to their capacity to adapt their structure and bind to a variety of different interaction sites [58,59]. In a prior study, we identified a SLiM which we coined as the ΩXaV motif (^261^FVEV^264^) within RVFV NSs, demonstrated that this motif was crucial for the interaction between NSs and the p62 subunit of the general host transcription factor TFIIH [11], and that the aromatic residue F261 played a crucial role in the complex formed between these two proteins. In particular, these studies demonstrated that a F261S substitution within the NSs protein disrupted its function in RVFV infected cells including reducing NSs-dependent filament formation as well as decreasing RVFV infectious titers [11]. Our current studies have determined that these same amino acids can function as an LIR motif and enable NSs to form interactions with the human LC3-family proteins in RVFV infected cells. Structural analysis using X-ray crystallography indicates that at the atomic level F261 and V264 form multiple hydrophobic interactions with residues in the HP1 and HP2 of both LC3A and GABARAP, respectively. Interestingly, these two residues also formed key interaction between NSs and the p62 subunit of TFIIH, indicating the importance of this SLiM for multiple functions associated with the NSs protein of RVFV. The ability of NSs to interact with multiple proteins using this small stretch of amino acids (^261^FVEV^264^) highlights the versatile nature of NSs. It is possible that NSs interacts with different proteins using this same motif at distinct stages of viral replication to most efficiently subvert the host’s antiviral response. NSs induces degradation of TFIIH p62 at early time points after infection (e.g. 2–4 hours) [10,12,53]. Our current studies showed the impact of NSs on autophagy at 8 and 24 hpi and NSs-LC3 interaction at 24 hpi, suggesting the NSs interaction with LC3 and inhibition of autophagy are important for later stages of replication. However, a more detailed time course analysis would be needed to elucidate the timing of NSs-LC3 interactions in comparison to NSs-TFIIH p62 interactions.

Our results provide the first mechanistic insight into how RVFV uses its main virulence factor NSs to regulate autophagy during an infection. LC3 family members are key mediators of autophagosome formation and elongation in the cytoplasm [60]. NSs was found to interact with all six human LC3-family members both *in vitro* and in RVFV-infected cells. The interaction required F261, as reduced binding between NSs and LC3A and LC3B was observed in infected cells expressing a variant of NSs where F261 was substituted by a Ser residue (F261S). Our results also indicate that the interaction between NSs and LC3 proteins occured predominantly in the nucleus and perinuclear regions, and that LC3 co-localized with NSs within the nuclear filaments formed by NSs during RVFV infection. The presence of LC3A in the NSs filaments is consistent with the ability of NSs to sequester other host proteins within these filaments, including SAP30 and TFIIH p44 [9,10]. Furthermore, LC3A was found to localize primarily in the nucleus of RVFV infected cells, whereas it was primarily located in the cytoplasm of infected cells expressing the F261S variant of NSs. NSs F261S was more predominantly located in the cytoplasm, but the mechanism behind this altered distribution is currently unknown. The molecular events leading to NSs nuclear import are also poorly understood. NSs does not contain a classical nuclear localization sequence, but loss of nucleoporin 98 (Nup98) reduced NSs nuclear localization [61]. Regardless of both NSs F261S and LC3A having a predominant cytoplasm localization, NSs F261S had a reduced ability to interact with LC3, highlightling the importance of NSs F261 for binding to LC3.

It has been established that important quantities of LC3-family proteins are present in nuclear stores under nutrient rich conditions and they are then transported to the cytoplasm upon autophagy initiation due to conditions such as serum starvation [49,62]. LC3 proteins do not contain a known nuclear localization sequence and it has been suggested that they enter the nucleus by means of passive diffusion [63]. Thus, our findings that LC3A is primarily nuclear in RVFV infected cells suggests that NSs functions by retaining LC3A in the nucleus and this serves to dampen the antiviral role of the host autophagy pathway. Indeed, we found that serum starvation induced autophagy was significantly suppressed in RVFV infected cells and this suppression was relieved when NSs was replaced by the F261S variant protein that lacked a functional LIR motif required for binding to the LC3-family proteins. However, the mechanism by which NSs retains LC3 proteins in the nucleus is yet to be determined and future studies will focus on elucidating mechanisms of nuclear retention of LC3 during RVFV infection.

Autophagy is a conserved host process that is often used by the immune system to help clear out viruses and viral particles [23], but it has also been shown that this system can be hijacked by viruses to facilitate their replication [64]. Following RVFV infection, autophagy induction has been observed in several types of mammalian cells and the impact on the virus is dependent on the cell type [18,19]. Herein we found that that NSs F261S results in a substantial decrease in NSs binding to LC3 family members and significantly reduces the ability of RVFV to block autophagy during infection. Interestingly, NSs F261S is also deficient in TFIIH p62 binding and degradation [11] and RVFV NSs suppresses interferon partially through TFIIH p62 degradation [10,12,53]. rMP-12 NSs-3XFlag F261S replicates less efficiently in interferon competent HSAECs as compared to rMP-12 NSs-3XFlag. However, similar replication was observed for both viruses in interferon deficient Vero cells; indicating that the lack of an interferon response can rescue the attenuated F261S phenotype. These results were not unexpected given the significant cross-talk between autophagy and interferon mediated signaling [23,54]. Autophagy can aid in viral clearance through degradation of viral components, but viral recognition is enhanced during this process through pattern recognition receptors, such as Toll like receptor 7 (TLR7), contributing to interferon induction [55–57]. Therefore the ability of NSs F261 to suppress autophagy provides another mechanism by which RVFV NSs can suppress innate immune signaling. Future work will determine the importane of NSs F261 for RVFV pathogenesis in mouse models.

## Materials and methods

### Alpha-fold analysis

A computational pipeline was assembled for *in silico* binding studies of NSs with the six human LC3-family proteins. In brief, AlphaFold [34,35] pre-trained multimer models were used to fold NSs in the presence of each of the six LC3-family proteins. Each of the 5 pre-trained Alpha-fold models was run with 5 random seeds creating 25 total models with distinct binding structures. FoldX [36] was applied to the resulting models to refine the positions. A series of biophysical parameters calculated from these structures was used to evaluate their quality including total energy in kcal/mol, and accessible surface area of the LIR motif before and after binding [37]. These parameters allowed for ranking the interactions of the models on binding specificity and energy. Similarly, the resulting models were inspected using UCSF Chimera [65] to verify the binding mode of the LIR motifs of NSs with each LC3-family member and to compare with other available LIR-target protein structures from the RCSB Protein Data Bank [66].

### Expression vectors

The sequences encoding for LC3A (residues 1–121 of human LC3A), LC3B (residues 1–125 of human LC3B), LC3C (residues 8–125 of human LC3C), GABARAP (residues 1–117 of human GABARAP), GABARAPL1 (residues 1–117 of human GABARAPL1), GABARAPL2 (residues 1–117 of human GABARAPL2), NSs1 (residues 2–16 of RVFV NSs), NSs2 (residues 53–72 of RVFV NSs), NSs3 (residues 234–255 of RVFV NSs), NSs4-LC3A (residues 256–265 of RVFV NSs fused to LC3A) and NSs4-GABARAP (residues 256–265 of RVFV NSs fused to GABARAP) were synthesized as oligonucleotides (Integrated DNA Technologies) with flanking BamHI and EcoRI restriction enzyme sites and cloned into a modified pGEX-2T vector (Amersham) with a Tobacco Etch Virus (TEV) protease cleavage site replacing the original thrombin cut site. The vectors for expressing NSs4 (residues 247–265 of RVFV NSs), NSs F261S (NSs4 with Ser substituted for Phe at residue 261), and NSs4 V264S (NSs4 with Ser substituted for Val at residue 264 have been described previously [11,67]. For expressing EGFP-LC3A, EGFP-LC3B, EGFP-LC3C, EGFP-GABARAP, EGFP-GABARAPL1 and EGFP-GABARAPL2 in cells, the sequences encoding for the human sequences were inserted into the pEGFP-C1 vector using the BglII/EcoRI restriction sites and then transferred into the pCDNA3.1 vector. All constructs were verified by DNA sequencing.

### Peptides and protein expression and purification

LC3A, LC3B, LC3C, GABARAP, GABARAPL1, GABARAPL2, NSs4-LC3A, NSs4-GABARAP, NSs1, NSs2, NSs3, NSs4, NSs4 F261S and NSs4 V264S were expressed as GST-fusion proteins in *E*. *coli* host strain TOPP2. Cells were grown in Luria Broth (LB) medium supplemented with 100 mg/mL ampicillin overnight at 37°C. The next day, cells were diluted in LB plus antibiotics and grown until reaching O.D.600 between 0.6–0.8. Protein expression was induced with 1 mM IsoPropyl-b-D-ThioGalactopyranoside (IPTG; Inalco) for 4 h at 30°C. Cells were pelleted by centrifugation (15 min, 12,000 x g) and resuspended in lysis buffer (20 mM Tris-HCl pH 7.4, 1M NaCl, 0.2 mM EDTA, 1 mM DTT) and lysed using a French Press. The resulting suspension was centrifuged (1h, 105,000 x g, 4°C) and the supernatant incubated (1 h, 4°C) with a Glutathione Sepharose 4B (GSH; Cytiva) resin. The resin was then rinsed three times with TEV buffer (20 mM NaH2PO4/Na2HPO4 pH 7.4, 125 mM NaCl, 5 mM DTT) and incubated overnight at room temperature with the TEV protease. The next day, the proteins were further purified by ion exchange chromatography using either a High Performance SP-Sepharose (Cytiva) column (LC3A, LC3B, LC3C, GABARAP, GABARAPL1, GABARAPL2, NSs4-LC3A and NSs4-GABARAP) or a High Performance Q-Sepharose (Cytiva) column (NSs1, NSs2, NSs3, NSs4, NSs4 F261S, NSs4 V264S). For the ion-exchange chromatography, the columns were equilibrated in 20 mM sodium phosphate buffer pH 6.5 with 1 mM DTT (buffer A) and the proteins eluted with a gradient of 20 mM sodium phosphate buffer pH 6.5 with1 M NaCl and 1 mM DTT (buffer B). The fractions containing the protein from the ion exchange columns were then dialyzed into 20 mM sodium phosphate buffer pH 6.5 with 50 mM NaCl and 1 mM DTT and purified by gel filtration chromatography over a Sepharose 12 10/300GL column (GE Healthcare) and the final purified proteins stored at -80°C prior to usage in ITC and crystallography experiments.

### NMR experiments

For the NMR chemical shift perturbation experiments with the LC3 proteins, unlabeled NSs4 was added to 0.5 mM of either ^15^N-labeled LC3A or ^15^N-labeled LC3B to a final ratio of 1:1.5. For NMR chemical shift perturbation experiments with NSs4, unlabeled LC3A was added to 0.5 mM ^15^N-labeled NSs4 to a final ratio of 1:1.5. To map the binding site of Nss4 on the LC3B protein, the backbone assignment of LC3B was obtained from the Biological Magnetic Resonance Data Bank (accession numbers 26881) and the amino acids of LC3B showing a significant chemical shift change were determined (Δδ(ppm) > 0.15; Δδ = [(0.17ΔN_H_)^2^ + (ΔH_N_)^2^]^1/2^} and mapped on the structure of LC3B (PDB code 3VTU). The NMR data were processed with NMRPipe/NMRDraw and analyzed with CcpNMR. All NMR experiments were carried out at 300 K on a 4-channel Bruker BioSpin 700-MHz spectrometer in 20 mM sodium phosphate (pH 6.5), 50 mM NaCl and 90% H_2_O, 10% D_2_O.

### ITC experiments

Proteins were dialyzed overnight at room temperature into 20 mM sodium phosphate buffer pH 6.5 with 50 mM NaCl. Protein concentrations were determined by UV absorbance at 280 nm. ITC measurements were performed at 25°C using a VP-ITC calorimeter (MicroCal). Data were analyzed using Origin Software and all experiments fit the single binding site model with 1:1 stoichiometry. Standard deviations in *K*_D_ values were determined from duplicate measurements or more.

### Crystallization and data collection

NSs4-LC3A and NSs4-GABARAP fusion proteins were dialyzed into 25 mM MES, 50 mM NaCl with 1mM DTT buffer and used at concentrations varying between 150–700 μM in 25 mM MES, 50 mM NaCl and 1mM DTT buffer. For crystallization of GABARAP with the NSs3 peptide, the NSs3 peptide was added to give a protein:peptide ratio of 1:2. The crystals of the fusion proteins and the protein:peptide complexes were generated using the hanging drop vapor diffusion method. The selected crystals following screening of varying conditions were cryoprotected in a mother liquor containing 20% glycerol. Diffraction data collected using Pilatus3S 6M at beamline 08-ID of the Canadian Light Source (CLS) or either a Pilatus3S 6M detector or at the beamline IDB7 of the Macromolecular Cornell High Energy Synchrotron Source (MacCHESS). Datasets were indexed, integrated and scaled using HKL2000 (HKL Research, Inc.).

### Structure determination and refinement

For calculation of the structures from the crystal data, the initial phases were generated by molecular replacement using the crystal structure of LC3A (PDB:3WAL) and GABARAP (PDB:1GNU) as a search template. The final phases were determined following iterative cycles of model building with Coot and refinement using PHENIX [68]. Test data sets were selected randomly from the observed reflections prior to refinement. Statistics for the final models were obtained with PHENIX and Molprobity, and the figures were prepared with PyMOL [69].

### Cell culture

Vero cells (African green monkey kidney epithelial cells) were acquired from American Type Culture Collection (ATCC, CCL-81), and were maintained in Dulbecco’s modified minimum essential medium (DMEM) supplemented with 10% fetal bovine serum (FBS), 1% L-glutamine, and 1% penicillin/streptomycin. Human small airway epithelial cells (HSAECs) were acquired from Cambrex Inc., Walkersville, MD and maintained in Ham’s F12 medium supplemented with 10% FBS, 1% L-glutamine, 1% penicillin/streptomycin, 1% nonessential amino acids, 1% sodium pyruvate, 0.001% of 55 mM β-mercaptoethanol. Baby hamster kidney cells, which stably express T7 DNA-dependent RNA polymerase (BSR-T7/5), were maintained in minimal essential medium (MEM) supplemented with 7.5% FBS, 1% penicillin/streptomycin, and 1 mg/mL Geneticin (Gibco, G418) [70]. Baby hamster kidney cells (BHKs) were cultured in Modified Eagle Medium (MEM) supplemented with 10% fetal bovine serum (FBS), 1% L-glutamine, and 1% penicillin/streptomycin. All cell lines were maintained at 37°C with 5% CO_2_.

### Viruses

Production of RVFV recombinant (r)MP12 was performed via transfection of BSR-T7/5 cells (seeded at 1x10^6^ cells per 25cm^2^ flask) utilizing six plasmids: pProT7-vL(+), pProT7-vM(+), pProT7-vS(+), pT7-IRES-N, pCAGGS-G, and pT7-IRES-L [10,71]. To generate an initial seed stock, BSR-T7/5 cells were transfected with 2.0 μg each of pProT7-vL(+), pProT7-vM(+), pProT7-vS(+)((r)MP12) or pPro-T7-vS-F261S(+) (rMP-12 F261S), pT7-IRES-N and 1.0 μg of pCAGGS-G, pT7-IRES-L using TransIT-LT1 (Mirus, 2300). The ratio of total plasmid DNA amount (μg) to TransIT-LT1 volume (μL) was 1:3. Complete media (cMEM) without geneticin selection was utilized during transfection and subsequent culturing. At 24 h post-transfection (hpi), media was removed, cells rinsed once with cMEM, and cMEM without geneticin was added back. After an additional 72 h, media supernatants were collected, filtered (0.2 μm), aliquoted, and stored at -80°C. Infectious viral titers were determined by plaque assay on Vero or BHK cells as previously described [72]. To generate a passage one (P1) viral working stock, Vero cells (seeded at 1x10^7^ per 175cm^2^ flask) were infected at an MOI of 0.1. cDMEM was added and cells cultured for 48 h. Media supernatants were collected, filtered (0.2 μm), aliquoted, and stored at -80°C. Viral titer was determined via plaque assay on Vero cells.

RVFV rMP-12 NSs-3XFlag and RVFV rMP-12 NSs-3XFlag F261S, which contain a triple tandem flag tag on the NSs protein, were generated as previously described [11]. These viruses were rescued following the described protocol for the (r)MP12 above.

### Antibodies

Antibodies specific for LC3A (4599), LC3B (3868), GAPDH (5174), and Lamin A/C (4777) were obtained from Cell Signaling Technology (Beverley, MD). The antibody specific for monoclonal Anti-Flag (F1804) was obtained from Millipore Sigma (Rockville, MD). Antibodies specific for HRP Anti-beta actin (ab4900) and Green florescent protein (GFP) (ab290) were obtained from Abcam (Waltham, Boston). Secondary antibodies specific for Goat anti-mouse IgG (32430) and Goat anti-Rabbit IgG (32460) were obtained from ThermoFisher Scientific (Rockford, IL).

### LC3 family member transfections

BSR-T7/5 cells were transfected with GFP-LC3-family member plasmids (pCDNA3.1-EGFP/LC3A, pCDNA3.1-EGFP/LC3B, pCDNA3.1-EGFP/LC3C, pCDNA3.1-EGFP/GABARAP, pCDNA3.1EGFP/GABARAPL1, and pCDNA3.1-EGFP/GABARAPL2) or GFP control (pCDNA3.1-EGFP) using TransIT-LT1 transfection reagent (Mirus, 2300) according to the manufactory’s instructions. Briefly, 2.5 μg plasmid DNA, 250 μL Opti-MEM I Reduced-Serum Medium, and 7.5 μL warmed TransIT-LT1 Reagent was added to microcentrifuge tubes and mixed, then incubated for 30 min at room temperature. BSR-T7/5 cells (seeded at 2.5x10^6^ per 75cm^2^ flask) were transfected with TransIT LT1 reagent DNA complex via adding the mixture dropwise and rocking to distribute the plasmids. The flasks were imaged 24 h post transfection using an EVOS M5000 Imaging System (ThermoFisher, AMF5000) to visualize GFP expression and confirm successful transfection.

### Viral infections

HSAECs and Vero cells (seeded at 2.5x10^6^ per 75cm^2^ flask) were infected at MOI 3 with (r)MP-12, rMP-12 NSs-3XFlag, rMP-12 NSs-3XFlag F261S, or mock-infected (media only, negative control) for 1 h, then the infection media was replaced with fresh HSAEC or Vero media for 24 h. Cells were collected at 24 hpi for analysis.

BSR-T7/5 cells (seeded at 0.3x10^6^ per well in a 6 well plate) were transfected as described above and infected at MOI 0.1 with rMP-12 NSs-3XFlag 24 h post transfection. Cells were collected at 24 hpi for analysis.

### Immunoprecipitation

Cells underwent trypsinization (Corning, 25-053-CI), then were resuspended in cell culture media, and subjected to centrifugation (2,500 rpm, 10 min). The supernatant was discarded, pellets were resuspended in 1mL sterile PBS (ThermoFisher, 10010023), and samples were centrifuged (2,500rpm, 10 min). PBS was then discarded and pellets were resuspended vigorously in clear lysis buffer (50 mM Tris-HCl pH 7.4, 120 mM NaCl, 5 mM EDTA, 0.5% NP-40, 50 mM NaF, 0.2 mM Na3VO4, and EDTA-free complete protease inhibitor cocktail (Roche, 11697498001)) based on flask size (300 μL per 75cm^2^ flask, 500 μL per 175cm^2^ flask, 1 mL per 225 cm^2^ flask). Samples were incubated on ice and vortexed every 5 min for 20 min. Following incubation, samples were subject to centrifugation (15,000 rpm, 10 min). Pellets were discarded and lysates were quantified via Bradford reagent (ThermoFisher, 23236). One mg of protein was used for each IP sample; the sample volume was balanced with clear lysis buffer for a total sample volume of 250 μL. Input samples were collected as 10% of the IP sample volume. HSAECs and Vero cell IP samples were incubated with 1 μg of LC3A or LC3B antibody and BSR-T7/5 IP samples were incubated with 1μg GFP antibody and samples were incubated overnight rotating at 4°C. The next day, Protein G Dynabeads (Invitrogen, 10003D) were prepared by obtaining 50 μL beads per sample, washing twice with citrate phosphate buffer (0.5M, pH 5.0; 1L DI water, 18.15g of Sodium Phosphate Dibasic Dihydrate, 9.605 g of Citric Acid), twice with PBS, and resuspension in PBS (50 μL per sample). Fifty μL of the prepared Protein G beads was added to each IP sample and they were incubated at room temperature rocking for 45 min. After the rotation, samples were washed with 500 μL of TNE150 (50 nM Tris-HCl (pH 7.5), 150 mM NaCl, 1 mM EDTA, and 0.1% NP-40), 500 μL of TNE 50 (50 nM Tris (pH 7.5), 150 mM NaCl, 1 mM EDTA, and 0.1% NP-40), and 250 μL of PBS. All washes were discarded and samples were eluted in blue lysis buffer (25 μL per IP sample and 1:1 ratio for input samples). Blue lysis buffer is composed of 25 mL 2x Novex Tris-Glycine Sample Loading Buffer SDS (Invitrogen, LC2676), 20 mL T-PER Tissue Protein Extraction Reagent (Thermo Scientific, 78510), 200 μL 0.5 M EDTA pH 8.0, EDTA-free complete protease inhibitor cocktail (Roche, 11697498001), 80 μL 0.1 M Na3VO4, 400 μL 0.1M NaF, and 1.3 mL 1M DTT. Then samples were subjected to western blot protocol.

### Cellular fractionation

HSAECs were seeded at 2.5x10^6^ per 225cm^2^ flask and incubated overnight. Cells were then infected with media only (mock), rMP-12 NSs-3XFlag, or rMP-12 NSs-3XFlag F261S at an MOI 3 for 1 h. At 24 hpi, cell suspension containing 5x10^6^ cells was transferred to 15 mL conical tubes for each condition, respectively. The Qiagen Qproteome® Cell Compartment Kit (Qiagen, 37502) Subcellular Fractionation of Cultured Cell Samples Protocol was then meticulously followed. Following fractionation, the produced extracts were quantified using Bradford reagent (ThermoFisher, 23236) and checked for purity via western blot probing for GAPDH (cytoplasmic) and Lamin A/C (nuclear). Fractions underwent the described immunoprecipitation protocol followed by western blot analysis probing for Flag and LC3B.

### Western blot

Samples were collected and prepared as described in the co-immunoprecipitation or cellular fractionation protocol. Samples were separated on 4–12% bis-tris gels, wet transferred for 1 h on PVDF transfer membranes (VWR, 490007) and blocked for 1h at room temperature with 5% bovine serum albumin (BSA) (ThermoFisher, B14) in Tris-buffered saline solution containing 0.1% Tween (TBST). Membranes were probed with primary Anti-Flag, anti-LC3A, anti-LC3B, anti-GFP, GAPDH, Lamin A/C, or beta-Actin antibodies, which were diluted in 5% BSA blocking buffer and incubated overnight on a rocker at 4°C. The membranes were washed with tris buffered saline (TBS), TBST, TBS for 5 min each, incubated for 1h at room temperature rocking with secondary anti-rabbit (LC3A, LC3B, GFP) or secondary anti-mouse (Flag) diluted in blocking buffer (1:1000), then washed with TBS, TBST, TBST, TBS for 5 min each. The membranes were imaged via chemiluminescence using the SuperSignal West Femto Maximum Sensitivity substrate kit (Thermo Scientific, 34095) on the Bio Rad ChemiDoc MP Imaging System.

### Confocal microscopy

HSEACs and Vero cells were seeded on coverslips in separate six-well plates (1x10^5^ cells per well) and grown to 70% confluency. After 24 h, cells were infected with rMP-12 NSs-3XFlag (MOI 3.0) or rMP-12 NSs-3XFlag F261S (MOI 3.0). Mock infected cells (incubated with complete media) were included as a negative control. Infected and mock infected controls were collected at 24 hpi. As a positive control, uninfected cells were treated with serum starvation media (1% BSA, 140 mM NaCl, 1 mM CaCl2, 1 mM MgCl2, 5 mM glucose, and 20 mM HEPES [pH 7.4] at 37°C for 4 h in Vero cells and 8h in HSAECs under 5% CO2) [62]. At the time of collection, cells were washed with PBS (−Ca^2+^ and −Mg^2+^) and then fixed with 4% (wt/vol) neutral buffered paraformaldehyde. Fixed cells were permeabilized with 0.1% (vol/vol) Triton X-100 in PBS for 10 min at room temperature then washed three times with PBS. Cells were blocked in 1% bovine serum albumin and 0.025% Triton X-100 in PBS, washed three times with PBS and probed overnight with Flag antibody (1:1000) and LC3A antibody (1:100). After 24 h, cells were washed three times with 0.025% Triton X-100 in PBS and probed with Alexa Fluor 568 goat anti-mouse secondary antibody (1:1000; Invitrogen, 11004) and Alexa Fluor 488 goat anti-rabbit secondary antibody (1:1000; Invitrogen, 11008). DAPI (4’,6-diamidino-2-phenylindole, 1:1000) was used to visualize nuclei. Coverslips were mounted to glass slides using Fluoromount-G (Southern Biotech, 0100). Slides were imaged using a water-immersion 60× objective lens on a Zeiss LSM 880 confocal laser scanning microscope. Samples were imaged at least 10 times each across 3 biological replicates, with a representative image shown. LC3 fluorescent intensity in the nucleus and cytoplasm was quantified utilizing ImageJ FIJI software. To ensure that LC3 signal was representative of LC3 nuclear retention, a nuclear mask was created via the DAPI channel and applied to the LC3 channel. LC3 nuclear signal was quantified by measuring the LC3A intensity in the nucleus out of the total intensity of LC3A in the cell. The cytoplasmic area was then quantified via defining the region of interest and subtracting out the nuclear LC3 signal. Nuclear and cytoplasmic LC3 signal was quantified for 25 cells/condition across 3 biological replicates and corrected nuclear or cytoplasmic cell fluorescent was calculated respectively utilizing the formula Integrated Density-(Area*mean fluorescent of background reading).

### Autophagy assay

Vero cells were seeded on separate coverslips in separate six-well plates (1x10^5^ cells per well) and grown to 70% confluency. Cells were then washed with PBS three times and either mock, rMP-12 NSs-3XFlag, or rMP-12 NSs-3XFlag F261S infected (MOI 3.0) and collected at 8 or 24 hpi. Four hours prior to collection, cells were serum starved. Mock infected cells that were not subjected to serum starvation were included for both the 8 h and 24 h time point. Following collection, cells underwent the Abcam Autophagy Detection Kit Protocol (Abcam, ab139484) according to the manufacturer’s instructions, followed by imaging via confocal microscopy. Slides were imaged using a water-immersion 60× objective lens on a Zeiss LSM 880 confocal laser scanning microscope. At least 50 cells per condition were imaged, with a representative image shown. Average foci per cell were counted for 50 cells/condition manually, assisted by ImageJ FIJI Software.

The imaging settings are displayed in Table 4.

**Table 4:** Imaging settings for the autophagy microscopy analysis.

	FITC (488 nm)	DAPI (405 nm)
**8 h samples**		
Master Gain	750	750
Digital Gain	9.0	5.8
Digital Offset Background	33	12
Laser Intensity	8.0%	2.0%
**24 h samples**		
Master Gain	750	750
Digital Gain	10.0	7.0
Digital Offset Background	37	33
Laser Intensity	8.0%	4.5%

### Statistics

Statistical analysis was conducted using a one-way ANOVA and post-hoc sidak’s test via GraphPad Prism Software. ns = not significant (P > 0.05), * = P ≤ 0.05, ** = P ≤ 0.01, *** = P ≤ 0.001, **** = P ≤0.0001.

## Supporting information

S1 Fig RVFV NSs interacts with LC3 family members *in silico* via LIR motifs. Representative structural models of RVFV NSs (orange) in complex with GABARAPL1 and GABARAPL2 (cyan) and LC3B and LC3C (forest green) proteins, as calculated via AlphaFold and FoldX (Materials and Methods). The C-terminal LIR motif of NSs (i.e., NSs4; purple atoms) interacts with LC3 family members in all cases.
(TIF)

S2 Fig RVFV LIR motifs interacts with LC3 family members *in silico*. Representative structural models of LIR motif-containing peptides (orange) corresponding to NSs1-3 in complex with GABARAP (cyan) and LC3A (forest green) proteins, as calculated via AlphaFold and FoldX (Materials and Methods). The LIR motifs of NSs (purple atoms) interact with LC3A and GABARAP in a similar manner.
(TIF)

S3 Fig NMR mapping studies of NSs4 binding to LC3A and LC3B. (A) Overlay of 2D ^1^H-^15^N HSQC spectra of ^15^N-labeled LC3A in the absence (black) and presence of NSs4 (red); (B) Overlay of 2D ^1^H-^15^N HSQC spectra of ^15^N-labeled LC3b in the absence (black) and presence of NSs4 (red): (C) Ribbon model of the three-dimensional structure of LC3B (lime; PDB code 3VTU) highlighting the residues that undergo significant chemical shift changes in the presences of NSs4. The amino acids of LC3B showing a significant chemical shift change {Δδ(ppm) > 0.15; Δδ = [(0.17ΔN_H_)^2^ + (ΔH_N_)^2^]^1/2^} upon addition of NSs4 are colored in red, where ΔN_H_ and ΔH_N_ are the difference in chemical shift between the two signals in ppm: (D) Overlay of 2D ^1^H-^15^N HSQC spectra of ^15^N-labeled NSs4 in the absence (black) and presence of LC3A (red).
(TIF)

S4 Fig Key interactions at the binding interfaces of the NSs4-LC3A and NSs4-GABARAP complexes. (A) Close-up and metrics (in Å) of the NSs4-LC3A complex highlighting the side chains of amino acids from HP2 of LC3A (forest green) that make hydrophobic interactions (F52, V54, P55, V58, L63, I66) with the side chain of V264 of NSs4 (firebrick red) at the binding interface. (B) Close-up and metrics (in Å) of the NSs4-GABARAP complex highlighting the side chains of amino acids from HP2 of GABARAP (cyan) that make hydrophobic interactions (Y49, V51, L55, F60, L63, F60) with the side chain of V264 of NSs4 (firebrick red) at the binding interface. (C) Close-up and metrics (in Å) of the NSs4-LC3A complex highlighting additional key interactions at the binding interface between LC3A (forest green) and NSs4 (firebrick red). They include interactions between D259-R10, E263-H27, D265-K30, V262-K49 and V262-F52 of NSs4 and LC3A respectively. (D) Close-up and metrics (in Å) of the NSs4-GARARAP complex highlighting additional key interactions at the binding interface between GABARAP (cyan) and NSs4 (firebrick red). They include interactions between D259-H8, E263-R28, D259-K48, V262-K46 and V262-Y49 of NSs4 and GABARAP, respectively. The dashed lines (black) in panels A-D corresponds to the distance measurements given in the text for the key interactions at the interfaces of the complex.
(TIF)

S5 Fig **Crystal structure of NSs3 in complex with GABARAP:** (A) Cartoon representation of the co-crystal structure of the NSs3-GABARAP complex highlighting the side chains of NSs3 (magenta) when complexed with GABARAP (cyan). (B) Close-up and metrics (in Å) of the NSs3-GABARAP complex highlighting the side chains of amino acids from HP1 of GABARAP (cyan) that make either hydrophobic (Y5, I21, P30, I32, L50, F104), anion-π (E17) or cation-π (K48) interactions with the side chain of W238 of NSs3 (magenta) at the binding interface. (C) Close-up and metrics (in Å) of the NSs3-GABARAP complex highlighting the side chains of amino acids from HP2 of GABARAP (cyan) that make hydrophobic interactions (Y49, V51, L55, F60, L63, F60) with the side chain of V241 of NSs3 (magenta) at the binding interface. (D) Close-up and metrics (in Å) of the NSs3-GARARAP complex highlighting additional key interactions at the binding interface between GABARAP (cyan) and NSs3 (magenta). They include interactions between N236-K46, I239-K46, I239-Y49, P240-L50, P240-R28, P243-L50 and P244-Q59 of NSs3 and GABARAP, respectively. The dashed lines (black) in panels A-D corresponds to the distance measurements given in the text for the key interactions at the interfaces of the complex.
(TIF)

S6 Fig LC3A and NSs colocalize in RVFV infected HSAECs. (A) HSAECs were grown on coverslips and mock infected (media alone) or infected with rMP-12 NSs-3XFlag. Cells were fixed at 24 hpi for staining. The cells were stained for LC3A (green), NSs-Flag (red), and DAPI (nuclear—blue). (B) Colocalization of LC3A and NSs was found in perinuclear and nuclear regions.
(TIF)

S1 Table Biophysical parameters of in silico interactions between NSs LIR-containing peptides and LC3 family members. *Total energy of the complex formed between the LIR motif with the binding target LC3 family protein in kcal/mol. **Change in accessible surface area (angstroms squared) upon binding of the LIR motif to target LC3 protein. ***The AlphaFold seed experiment which produced the highest ranking complex out of 25 total models.
(DOCX)

S2 Table Data collection and refinement statistics for LC3A-NSs4, GABARAP-NSs4 and GABARAP-NSs3. Values in parentheses are for highest-resolution shell. Rsym = ∑ hkl ∑i|Ihkl,i − <Ihkl>, where Ihkl,i is the intensity of an individual measurement of the reflection with Miller indices hkl and Ihkl is the mean intensity of the reflection. Rwork = ∑ hkl||Fo|−|Fc|| / ∑ hkl |Fo|, where |Fo| is the observed structure-factor amplitude and |Fc| is the calculated structure-factor amplitude. Rfree is the R factor based on at least 500 test reflections that were excluded from the refinements. CLS (Canadian Light Source) and CHESS (Cornell High Energy Synchrotron Source). a, Reflection for Fo > 0. b, MolProbity analysis.
(DOCX)

S1 Data This file include values used to create the graphs witin the paper. (XLSX)
