# Supplementary figures and images for: An LIR motif in the Rift Valley fever virus NSs protein is critical for the interaction with LC3 family members and inhibition of autophagy
Source: PLoS Pathog. 2024 Mar 21;20(3):e1012093. doi: 10.1371/journal.ppat.1012093 (PMC10986958; doi:10.1371/journal.ppat.1012093)

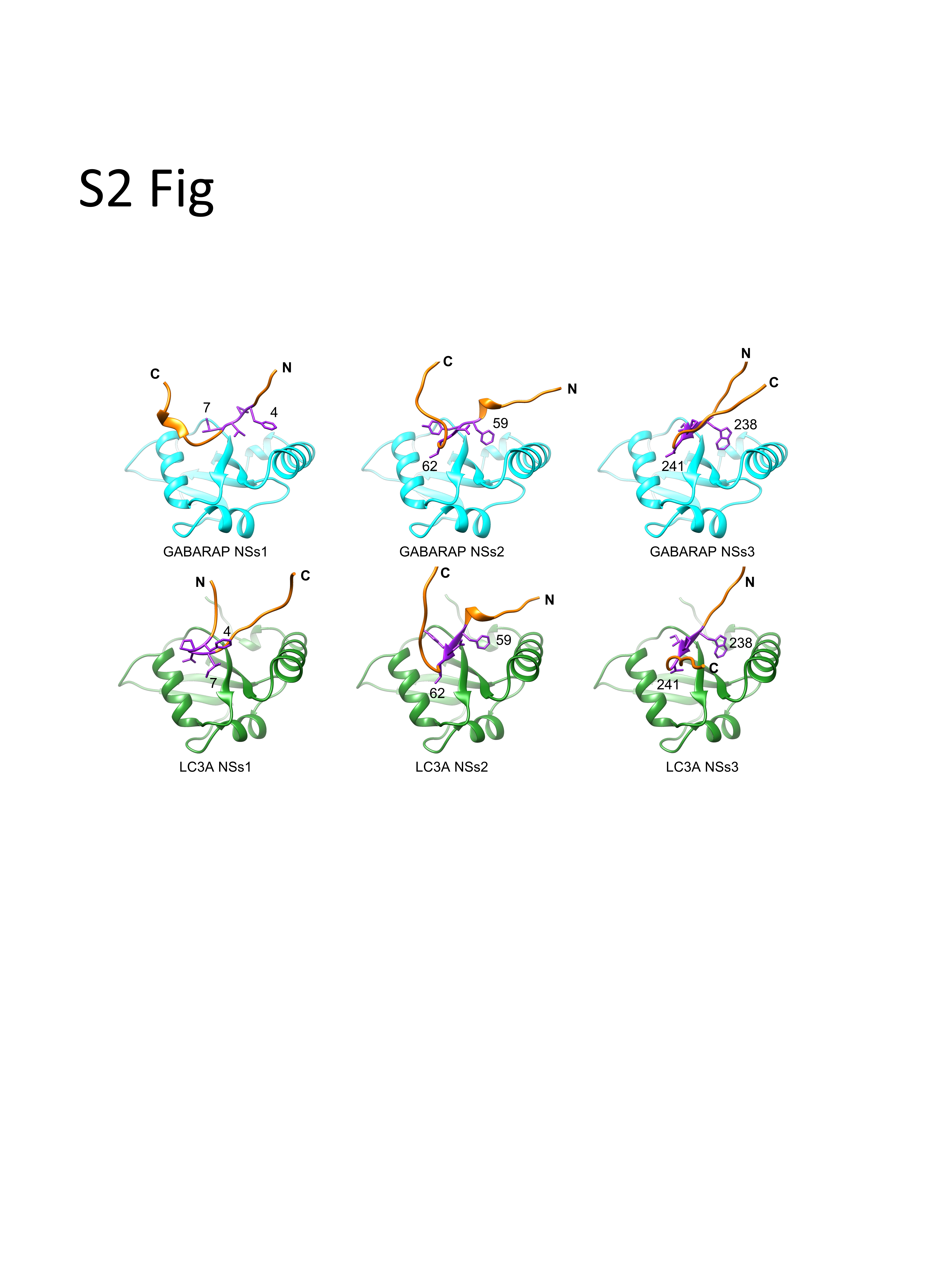

Supplement: S2 Fig — Representative structural models of LIR motif-containing peptides (orange) corresponding to NSs1-3 in complex with GABARAP (cyan) and LC3A (forest green) proteins, as calculated via AlphaFold and FoldX (Materials and Methods). The LIR motifs of NSs (purple atoms) interact with LC3A and GABARAP in a similar manner. (TIF) [file ppat.1012093.s002.tif]

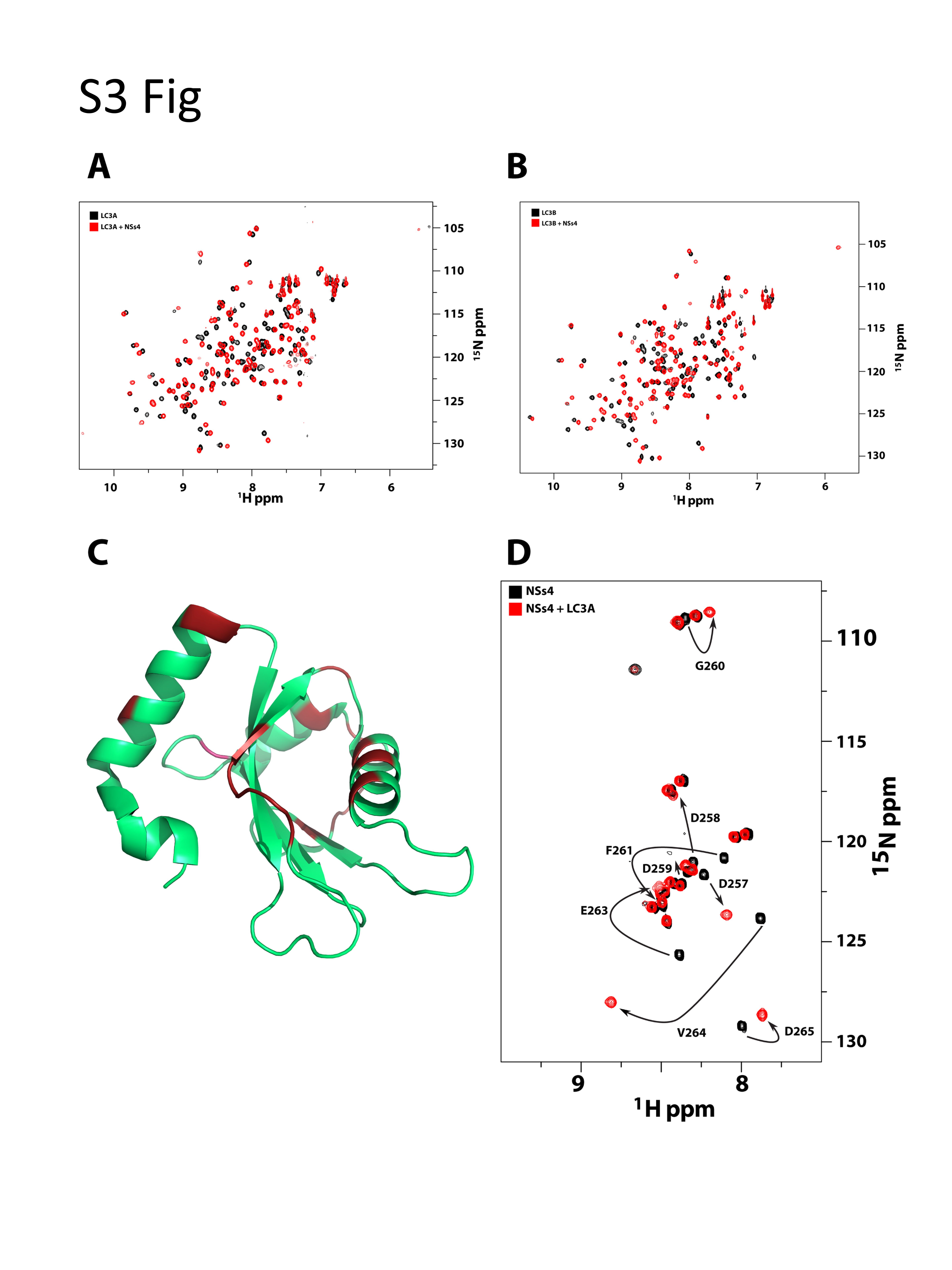

Supplement: S3 Fig — (A) Overlay of 2D 1H-15N HSQC spectra of 15N-labeled LC3A in the absence (black) and presence of NSs4 (red); (B) Overlay of 2D 1H-15N HSQC spectra of 15N-labeled LC3b in the absence (black) and presence of NSs4 (red): (C) Ribbon model of the three-dimensional structure of LC3B (lime; PDB code 3VTU) highlighting the residues that undergo significant chemical shift changes in the presences of NSs4. The amino acids of LC3B showing a significant chemical shift change {Δδ(ppm) > 0.15; Δδ = [(0.17ΔNH)2 + (ΔHN)2]1/2} upon addition of NSs4 are colored in red, where ΔNH and ΔHN are the difference in chemical shift between the two signals in ppm: (D) Overlay of 2D 1H-15N HSQC spectra of 15N-labeled NSs4 in the absence (black) and presence of LC3A (red). (TIF) [file ppat.1012093.s003.tif]

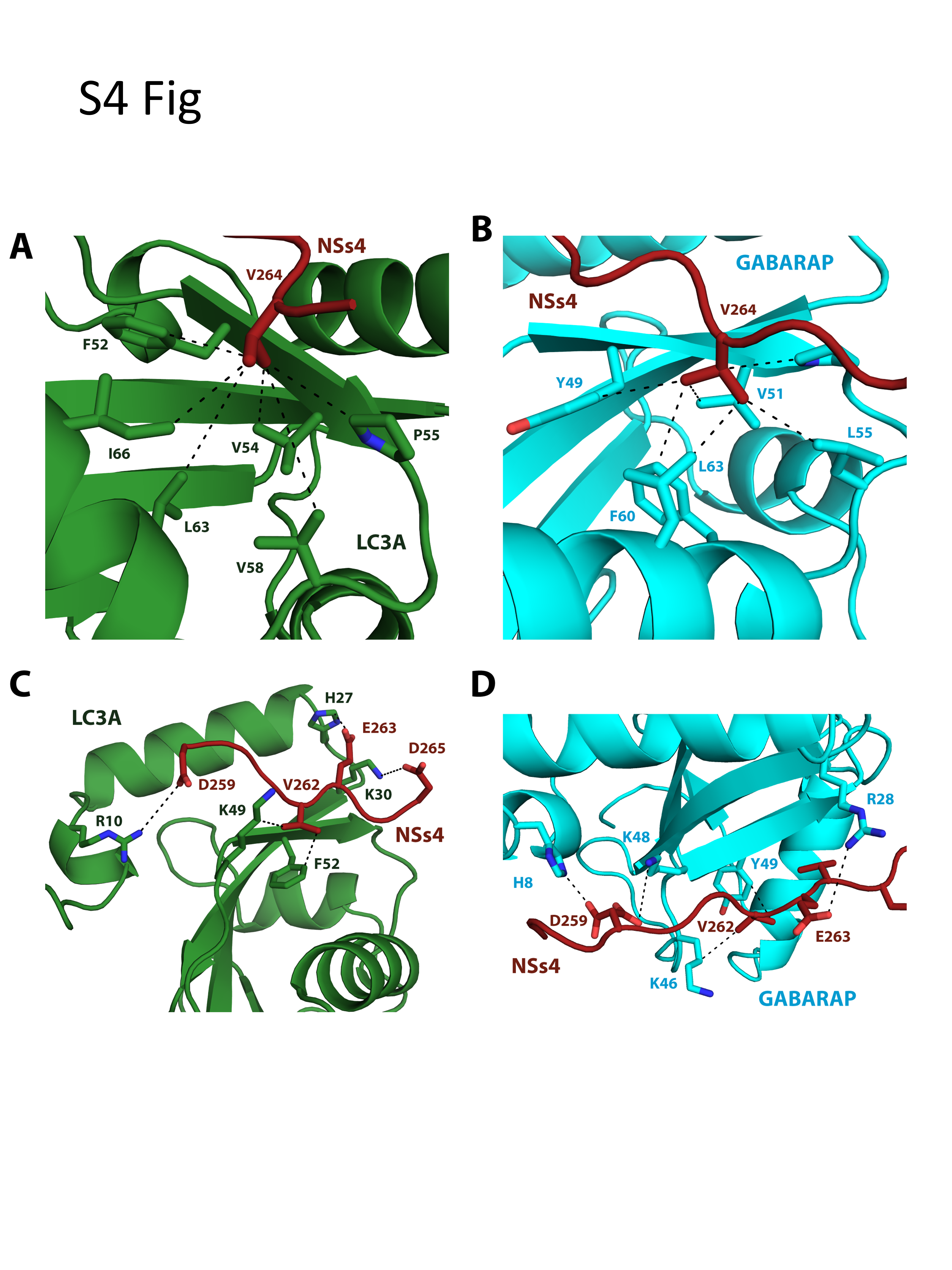

Supplement: S4 Fig — (A) Close-up and metrics (in Å) of the NSs4-LC3A complex highlighting the side chains of amino acids from HP2 of LC3A (forest green) that make hydrophobic interactions (F52, V54, P55, V58, L63, I66) with the side chain of V264 of NSs4 (firebrick red) at the binding interface. (B) Close-up and metrics (in Å) of the NSs4-GABARAP complex highlighting the side chains of amino acids from HP2 of GABARAP (cyan) that make hydrophobic interactions (Y49, V51, L55, F60, L63, F60) with the side chain of V264 of NSs4 (firebrick red) at the binding interface. (C) Close-up and metrics (in Å) of the NSs4-LC3A complex highlighting additional key interactions at the binding interface between LC3A (forest green) and NSs4 (firebrick red). They include interactions between D259-R10, E263-H27, D265-K30, V262-K49 and V262-F52 of NSs4 and LC3A respectively. (D) Close-up and metrics (in Å) of the NSs4-GARARAP complex highlighting additional key interactions at the binding interface between GABARAP (cyan) and NSs4 (firebrick red). They include interactions between D259-H8, E263-R28, D259-K48, V262-K46 and V262-Y49 of NSs4 and GABARAP, respectively. The dashed lines (black) in panels A-D corresponds to the distance measurements given in the text for the key interactions at the interfaces of the complex. (TIF) [file ppat.1012093.s004.tif]

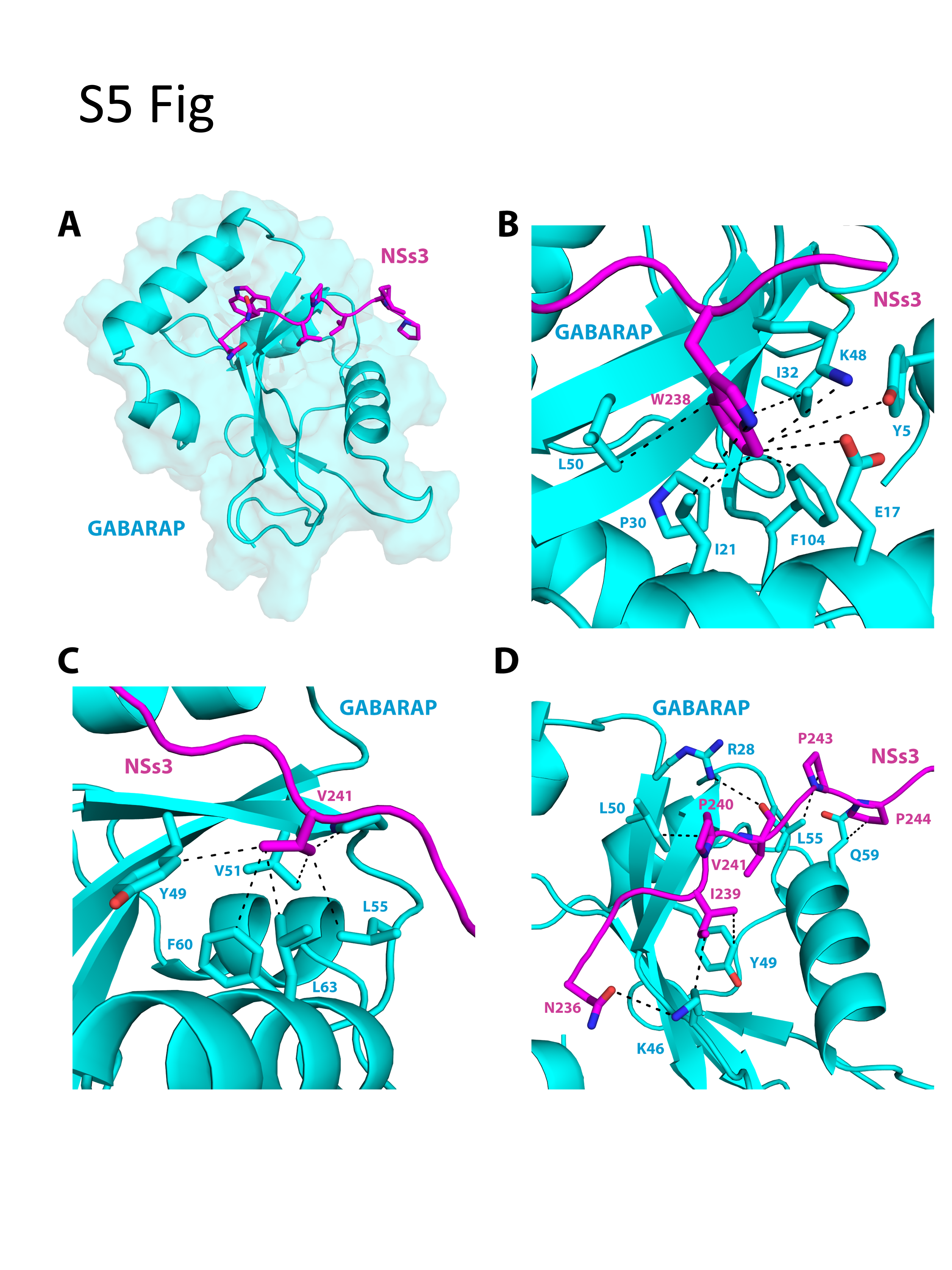

Supplement: S5 Fig — Crystal structure of NSs3 in complex with GABARAP: (A) Cartoon representation of the co-crystal structure of the NSs3-GABARAP complex highlighting the side chains of NSs3 (magenta) when complexed with GABARAP (cyan). (B) Close-up and metrics (in Å) of the NSs3-GABARAP complex highlighting the side chains of amino acids from HP1 of GABARAP (cyan) that make either hydrophobic (Y5, I21, P30, I32, L50, F104), anion-π (E17) or cation-π (K48) interactions with the side chain of W238 of NSs3 (magenta) at the binding interface. (C) Close-up and metrics (in Å) of the NSs3-GABARAP complex highlighting the side chains of amino acids from HP2 of GABARAP (cyan) that make hydrophobic interactions (Y49, V51, L55, F60, L63, F60) with the side chain of V241 of NSs3 (magenta) at the binding interface. (D) Close-up and metrics (in Å) of the NSs3-GARARAP complex highlighting additional key interactions at the binding interface between GABARAP (cyan) and NSs3 (magenta). They include interactions between N236-K46, I239-K46, I239-Y49, P240-L50, P240-R28, P243-L50 and P244-Q59 of NSs3 and GABARAP, respectively. The dashed lines (black) in panels A-D corresponds to the distance measurements given in the text for the key interactions at the interfaces of the complex. (TIF) [file ppat.1012093.s005.tif]

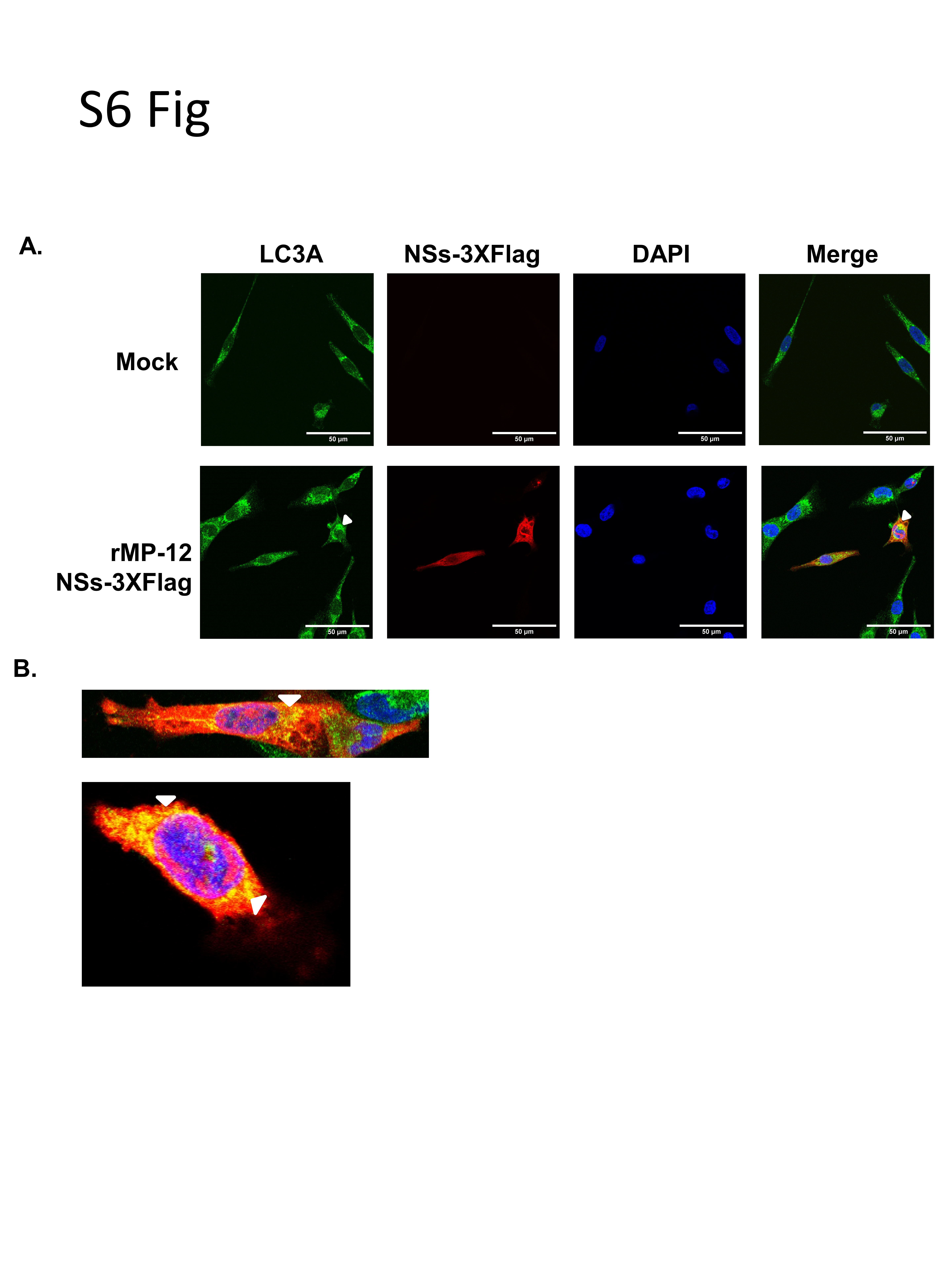

Supplement: S6 Fig — (A) HSAECs were grown on coverslips and mock infected (media alone) or infected with rMP-12 NSs-3XFlag. Cells were fixed at 24 hpi for staining. The cells were stained for LC3A (green), NSs-Flag (red), and DAPI (nuclear—blue). (B) Colocalization of LC3A and NSs was found in perinuclear and nuclear regions. (TIF) [file ppat.1012093.s006.tif]
