# Supplementary material for: An LIR motif in the Rift Valley fever virus NSs protein is critical for the interaction with LC3 family members and inhibition of autophagy
Source: PLoS Pathog. 2024 Mar 21;20(3):e1012093. doi: 10.1371/journal.ppat.1012093 (PMC10986958; doi:10.1371/journal.ppat.1012093)
Supplement: S1 Table — *Total energy of the complex formed between the LIR motif with the binding target LC3 family protein in kcal/mol. **Change in accessible surface area (angstroms squared) upon binding of the LIR motif to target LC3 protein. ***The AlphaFold seed experiment which produced the highest ranking complex out of 25 total models. (DOCX) [file ppat.1012093.s007.docx]

**S1 Table: Biophysical parameters of *in silico* interactions between NSs LIR-containing peptides and LC3 family members.**

| **RVFV NSs LIR** | **Binding Target** | **∆ Multimer ASA*** | **Binding Energy**** | **Rank***** |
| --- | --- | --- | --- | --- |
| NSs1 | GABARAP | 0.236 | -10.23 | 22 |
|  | GABARAPL1 | 0.261 | -10.57 | 23 |
|  | GABARAPL2 | 0.252 | -14.50 | 2 |
|  | LC3A | 0.260 | -10.90 | 4 |
|  | LC3B | 0.260 | -10.69 | 9 |
|  | LC3C | N/A | N/A | 0 |
| NSs2 | GABARAP | 0.272 | -20.61 | 1 |
|  | GABARAPL1 | 0.261 | -19.74 | 5 |
|  | GABARAPL2 | 0.289 | -21.19 | 13 |
|  | LC3A | 0.283 | -22.20 | 0 |
|  | LC3B | 0.278 | -24.89 | 20 |
|  | LC3C | 0.272 | -20.86 | 6 |
| NSs3 | GABARAP | 0.256 | -19.72 | 2 |
|  | GABARAPL1 | 0.271 | -20.74 | 1 |
|  | GABARAPL2 | 0.279 | -19.63 | 1 |
|  | LC3A | 0.266 | -14.88 | 8 |
|  | LC3B | 0.280 | -13.94 | 12 |
|  | LC3C | 0.257 | -16.18 | 0 |
| NSs4 | GABARAP | 0.399 | -17.78 | 17 |
|  | GABARAPL1 | 0.423 | -19.48 | 4 |
|  | GABARAPL2 | 0.423 | -17.74 | 11 |
|  | LC3A | 0.398 | -19.63 | 4 |
|  | LC3B | 0.423 | -24.23 | 12 |
|  | LC3C | 0.427 | -16.82 | 12 |

*Total energy of the complex formed between the LIR motif with the binding target LC3 family protein in kcal/mol.

**Change in accessible surface area (angstroms squared) upon binding of the LIR motif to target LC3 protein.

***The AlphaFold seed experiment which produced the highest ranking complex out of 25 total models.
