# Supplementary material for: An LIR motif in the Rift Valley fever virus NSs protein is critical for the interaction with LC3 family members and inhibition of autophagy
Source: PLoS Pathog. 2024 Mar 21;20(3):e1012093. doi: 10.1371/journal.ppat.1012093 (PMC10986958; doi:10.1371/journal.ppat.1012093)
Supplement: S2 Table — Values in parentheses are for highest-resolution shell. Rsym = ∑ hkl ∑i|Ihkl,i − , where Ihkl,i is the intensity of an individual measurement of the reflection with Miller indices hkl and Ihkl is the mean intensity of the reflection. Rwork = ∑ hkl||Fo|−|Fc|| / ∑ hkl |Fo|, where |Fo| is the observed structure-factor amplitude and |Fc| is the calculated structure-factor amplitude. Rfree is the R factor based on at least 500 test reflections that were excluded from the refinements. CLS (Canadian Light Source) and CHESS (Cornell High Energy Synchrotron Source). a, Reflection for Fo > 0. b, MolProbity analysis. (DOCX) [file ppat.1012093.s008.docx]

**S2 Table**: **Data collection and refinement statistics for LC3A-NSs4, GABARAP-NSs4 and GABARAP-NSs3**

| **Dataset** | **LC3A-NSs4** | **GABARAP-NSs4** | **GABARAP-NSs3** |
| --- | --- | --- | --- |
| **Data Collection** | | | |
| Beamline | F1, CHESS | **CMCF- BM,** CLS | ID7B2, CHESS |
| Wavelength (Å) | 0.97680 | 1.18057 | 0.96860 |
| Space group | P 2_1_ 2_1_ 2_1_ | P 2_1_ 2_1_ 2_1_ | C 2_1_ |
| **Unit cell parameters** | | | |
| a, b, c (Å) | 36.995 50.267 137.874 | 32.741 59.119 67.346 | 138.725 31.485 68.917 |
| α, β, γ (°) | 90, 90, 90 | 90 90 90 | 90 115.806 90 |
| Resolution range (Å) | 32.6 - 2.24 (2.32 -2.24) | 29.45 - 1.271 (1.316 - 1.271) | 36.74 - 1.88 (1.947 - 1.88) |
| No. of unique reflections | 12899 (1072) | 33702 (2064) | 20163 (833) |
| Multiplicity | 6.2 (5.9) | 11.3 (4.0) | 5.5 (2.3) |
| Completeness (%) | 93.28 (84.28) | 95.67 (59.89) | 88.41 (37.46) |
| R_merge_ | 0.09509 (0.7646) | 0.04741 (0.7465) | 0.05486 (1.77) |
| CC_merge_ | 0.997 (0.747) | 1 (0.567) | 0.999 (0.105) |
| I/σ(I) | 10.90 (2.45) | 20.69 (1.24) | 14.13 (0.31) |
| **Refinement Statistics** | | | |
| Resolution (Å) | 32.6 - 2.24 | 29.45 - 1.271 | 36.74 - 1.88 |
| Reflections (total/test)^[a](https://www.sciencedirect.com/science/article/pii/S096921261930437X?via%3Dihub#tblfn2)^ | 12130/1216 | 33690/1999 | 19732/1955 |
| R_work_/R_free_ (%) | 0.2319/0.2764 | 0.1539/0.1731 | 0.2205/0.2597 |
| CC_work_ | 0.928 (0.432) | 0.965 (0.470) | 0.956 (0.358) |
| CC_free_ | 0.914 (0.623) | 0.961 (0.639) | 0.906 (0.500) |
| **No. of atoms (excluding hydrogens)** | | | |
| Protein | 2094 | 1088 | 2189 |
| Water | 56 | 220 | 44 |
| **B factors** | | | |
| Protein | 57.47 | 17.35 | 63.66 |
| Water | 46.47 | 31.03 | 51.46 |
| **Root-mean-square deviation** | | | |
| Bond length (Å) | 0.002 | 0.009 | 0.002 |
| Bond angle (°) | 0.58 | 0.97 | 0.61 |
| **Ramachandran (%)****^[b](https://www.sciencedirect.com/science/article/pii/S096921261930437X?via%3Dihub#tblfn3)^** | | | |
| Favored | 96.37 | 99.21 | 97.23 |
| Outliers | 0.00 | 0.00 | 0.40 |

Values in parentheses are for highest-resolution shell. **R_sym_** = ∑ **hkl** ∑**i**|**I_hkl_**_,_**_i_** − <**Ihkl**>, where **I_hkl_**_,_**_i_** is the intensity of an individual measurement of the reflection with Miller indices **hkl** and **I_hkl_** is the mean intensity of the reflection. **R_work_** = ∑ **hkl**||**F_o_**|−|**F_c_**|| / ∑ **hkl** |**F_o_**|, where |**F_o_**| is the observed structure-factor amplitude and |**F_c_**| is the calculated structure-factor amplitude. **R_free_** is the [R factor](https://www.sciencedirect.com/topics/biochemistry-genetics-and-molecular-biology/r-factor) based on at least 500 test reflections that were excluded from the refinements. CLS (Canadian Light Source) and CHESS (Cornell High Energy Synchrotron Source).

a, Reflection for F_o_ > 0. b, MolProbity analysis.
